# FGF9 Recruits β‐Catenin to Increase Hepatic ECM Synthesis and Promote NASH‐Driven HCC

**DOI:** 10.1002/advs.202301166

**Published:** 2023-08-11

**Authors:** Lei Zhang, Qing Zhang, Da Teng, Manyu Guo, Kechao Tang, Zhenglin Wang, Xiang Wei, Li Lin, Xiaomin Zhang, Xiuyun Wang, Dake Huang, Cuiping Ren, Qingsong Yang, Wenjun Zhang, Yong Gao, Wei Chen, Yongsheng Chang, Huabing Zhang

**Affiliations:** ^1^ Department of Biochemistry and Molecular Biology Metabolic Disease Research Center School of Basic Medicine Anhui Medical University 230032 Hefei China; ^2^ Key Laboratory of Immune Microenvironment and Disease (Ministry of Education) Tianjin Key of Cellular Homeostasis and Disease Department of Physiology and Pathophysiology Tianjin Medical University 300070 Tianjin China; ^3^ Department of Hepatopancreatobiliary Surgery Affifiliated Chuzhou Hospital of Anhui Medical University (The First People's Hospital of Chuzhou) Chuzhou 239001 China; ^4^ Department of General Surgery The First Affiliated Hospital of Anhui Medical University 230022 Hefei China; ^5^ Synthetic Laboratory of School of Basic Medicine Sciences Anhui Medical University 230032 Hefei China; ^6^ Department of Microbiology and Parasitology School of Basic Medicine Anhui Medical University 230032 Hefei China; ^7^ Science and Technology Innovation Center Guangzhou University of Chinese Medicine 510006 Guangzhou China; ^8^ The Affiliated Chuzhou Hospital of Anhui Medical University (The First People's Hospital of Chuzhou) Chuzhou 239001 China

**Keywords:** extracellular matrix, FGF9, liver cancer, NASH, β‐catenin

## Abstract

Most nonalcoholic steatohepatitis (NASH) patients develop severe fibrosis through extracellular matrix (ECM) accumulation, which can lead to hepatocellular carcinoma (HCC). Fibroblast growth factor 9 (FGF9) is involved in serial types of cancer; however, the specific role of FGF9 in NASH‐driven HCC is not fully understood. This study finds that FGF9 is increased in patients with NASH‐associated HCC. Furthermore, NASH‐driven HCC mice models by feeding wildtype mice with high‐fat/high‐cholesterol (HFHC) diet and low dose carbon tetrachloride (CCl_4_) treatment is established; and identified that hepatic FGF9 is increased; with severe fibrosis. Additionally, AAV‐mediated knockdown of FGF9 reduced the hepatic tumor burden of NASH‐driven HCC mice models. Hepatocyte‐specific FGF9 transgenic mice (FGF9^Alb^) fed with a HFHC diet without CCl_4_ treatment exhibited an increased hepatic ECM and tumor burden. However, XAV‐939 treatment blocked ECM accumulation and NASH‐driven HCC in FGF9^Alb^ mice fed with HFHC diet. Molecular mechanism studies show that FGF9 stimulated the expression of ECM related genes in a β‐catenin dependent manner; and FGF9 exerts its effect on β‐catenin stability via the ERK1/2‐GSK‐3β signaling pathway. In summary, the data provides evidence for the critical role of FGF9 in NASH‐driven HCC pathogenesis; wherein it promotes the tumors formation through the ECM pathway.

## Graphical abstract

FGF9 induces non‐alcoholic steatohepatitis (NASH) to develop into severe fibrosis and eventually lead to hepatocellular carcinoma (HCC) by promoting extracellular matrix (ECM) accumulation. Mechanically, FGF9 exerts its effect on the regulation of the stability of β‐catenin via the ERK1/2‐GSK‐3β signaling pathway.

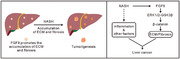

## 1 Introduction

Hepatocellular carcinoma (HCC) is the most common type of liver cancer and the end stage of chronic liver disease caused by different aetiologies, especially nonalcoholic steatohepatitis (NASH).^[^ ^1^ ^]^ The incidence of NASH‐driven HCC is expected to increase worldwide because of its association with the obesity and type 2 diabetes mellitus epidemic.^[^ ^2^, ^3^ ^]^ HCC has a poor prognosis, with frequent recurrence and metastasis. Although important improvements in the management of HCC have been made over the past decades, effective treatment options, such as local ablative therapies, resection or transplantation, are often limited to patients with early‐stage disease.^[^ ^4^ ^]^ Unfortunately, the therapeutic armamentarium for HCC is limited, ineffective and subject to inducing secondary or acquired chemoresistance via currently poorly understood mechanisms. Hence, there is an urgent need to understand HCC pathogenesis and identify new therapeutic targets.

Patients with NASH always exhibit extracellular matrix (ECM) accumulation and enhanced hepatic fibrosis.^[^ ^5^ ^]^ NASH‐induced chronic fibrosis has been shown to lead to HCC development. Consistent with a key structural and functional role of the ECM in liver tissues, recent reports have indicated that diet‐induced ECM accumulation fosters HCC development partially through the promotion of fibrosis.^[^ ^6^ ^]^ Aberrant accumulation of ECM has been implicated in fibrosis pathogenesis and progression, with a reduced capacity to compensate for increased adhesion and invasion, a key factor in hepatic cancer; however, the precise aetiology of this disease merits further investigation. Accumulating evidence suggests that therapeutically targeting the mechanisms leading to ECM dysfunction may have benefits for patients with liver disease.^[^ ^7^, ^8^, ^9^ ^]^ While ECM accumulation is an important characteristic of NASH, the exact mechanism leading to excessive accumulation of ECM in the liver remains unclear.

Recently, it has been shown that ECM accumulation in the liver requires ectopic induction of a Wnt signaling program that is normally associated with fibrosis.^[^ ^10^, ^11^, ^12^ ^]^ In the canonical Wnt pathway, Wnt ligands bind to the frizzled receptor and Fz‐coreceptors low‐density lipoprotein receptor‐related proteins (LRP) coreceptors, resulting in accumulation of β‐catenin in the nucleus. The activity of β‐catenin has emerged as a central determinant of ECM synthesis in the liver through its transcriptional control of the key genes of the ECM signaling pathway to facilitate ectopic ECM deposition in the liver.^[^ ^13^ ^]^ Therefore, a better understanding of the steps involved in the regulation of hepatic ECM accumulation might yield novel information regarding the pathogenesis of NASH‐driven HCC as well as identify potential targets for its treatment and prevention.

Fibroblast growth factor 9 (FGF9) is a paracrine hormone that regulates many physiological and pathological processes. It has been reported that FGF9 is a critical regulator of organ development.^[^ ^14^, ^15^ ^]^ In addition, the oncogenic roles of FGF9 in tumors have been characterized in previous studies.^[^ ^16^, ^17^, ^18^ ^]^ However, the contribution of FGF9 to NASH‐driven HCC remains unclear. In the current study, we explored a novel function of FGF9 in NASH‐driven HCC.

## 2 Results

### 2.1 Livers with NASH‐Driven HCC Exhibit Severe Fibrosis and Increased FGF9 Expression

**Figure 1:**
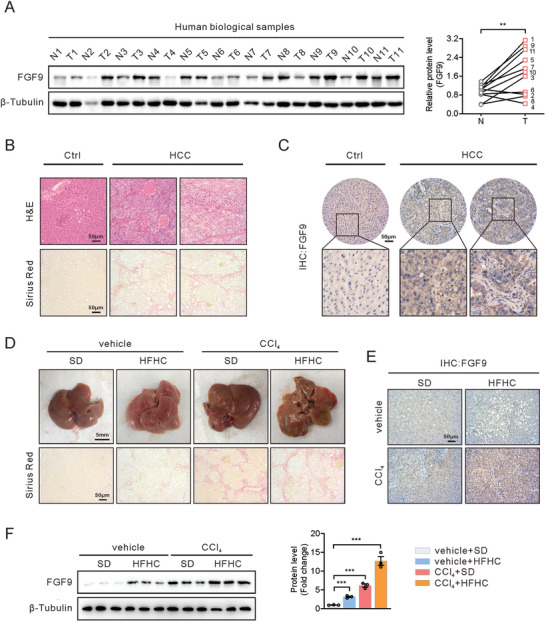
Increased FGF9 expression in human HCC samples and NASH‐driven HCC mice model. A) Western blot analysis of FGF9 in HCC and paired benign adjacent tissues (*n* = 11/group) (left panel) and quantification of FGF9 protein bands using Image J software (right panel). B) Hematoxylin‐eosin (H&E) (top) and Sirius Red (bottom) staining of HCC and paired benign adjacent tissues. C) Representative immunohistochemical (IHC) expression of FGF9 in HCC and benign adjacent tissues. D) Morphology (top) and Sirius Red (bottom) staining of liver sections from SD‐ and HFHC diet‐fed mice treated with or without CCl_4_. E) IHC analysis of FGF9 expression in liver nodules from D. F) Western blot analysis of FGF9 expression in livers from SD‐fed, HFHC diet‐fed, CCl_4_‐treated and HFHC diet and CCl_4_‐treated mice. All the data are presented as the means ± SEM. **p<0.01, ***p<0.005; 2‐tailed Student's *t* test (A), 2‐way ANOVA (F).

We previously confirmed that FGF9 expression is increased in severe fatty liver, although it is relatively low in normal liver tissue, suggesting that FGF9 may play an important role in regulating hepatic metabolic homeostasis.^[^ ^19^ ^]^ Therefore, we sought to determine whether FGF9 expression is induced in response to different stimuli that activate several metabolic processes in liver cells. In the present study, we explored the expression of FGF9 in NASH‐driven HCC. First, the expression of FGF9 was examined in a cohort of HCC patients exhibiting a distinct NASH phenotype. Liver samples from HCC patients showed higher FGF9 protein levels than samples from benign adjacent tissues by Western blot analysis (**Figure** **1**A). Moreover, histological examination further confirmed that the HCC slices showed severe NASH phenotypes, including steatosis and fibrosis (Figure 1B), accompanied with elevated FGF9 positive staining by using immunohistochemistry (IHC) analysis (Figure 1C).

Next, we explored the changes of FGF9 in the livers from the NASH‐driven HCC mouse models. It has been known that steatosis and fibrosis are characteristics in patients with NASH. The high‐fat/high‐cholesterol (HFHC) diet has been widely used to establish mouse NASH models because these dietary features is associated with NASH development in humans, and induces not only steatohepatitis, but also obesity and insulin resistance in mice.^[^ ^20^ ^]^ However, given that a HFHC diet alone does not induce fully progress to severe steatohepatitis and advanced fibrosis even after long‐term feeding.^[^ ^21^, ^22^, ^23^, ^24^ ^]^ Herein, we used relative low dose of carbon tetrachloride (CCl_4_; 20% in corn oil, 0.2 µL g^−1^ body weight), which did not induced the acute liver toxicity and excessive animal death,^[^ ^25^, ^26^, ^27^, ^28^ ^]^ to accelerate fibrosis and induce mouse NASH models.

Mice treated with HFHC showed significant weight gain and increased liver weight compared to standard diet (SD) feeding mice. CCl_4_ treatment attenuated the body weight gain and decreased liver weight induced by HFHC feeding (Figure S1B, Supporting Information). Whereas the SD or CCl_4_ mouse groups did not develop hepatic steatosis, accompany with increased cell proliferation capacity; however, both these parameters were increased in the two HFHC treated groups (HFHC or HFHC/CCl_4_) (Figure S1C, Supporting Information). The increased hepatic steatosis in HFHC or HFHC/CCl_4_ treated mice was accompanied by elevations of serum alanine aminotransferase (ALT), aspartate aminotransferase (AST), blood glucose, serum insulin and total serum cholesterol; in contrast to HFHC fed animals, these parameters were only modestly but not significantly increased by CCl_4_ treatment alone, indicating that while the NASH‐related histologic features were amplified by the addition of CCl_4_, but single CCl_4_ treatment has rare influence of NASH‐related histologic features, as reflected in mere changes of hepatic steatosis, hyperglycemia and hyperinsulinemia (Figure S1D, Supporting Information).

In addition, HFHC diet‐fed, CCl_4_‐treated mice exhibited spontaneous development of HCC, accompany with severe fibrosis (Figure 1D; Figure S1E, Supporting Information). Consistent with the increased HCC formation, the expression of markers involved in ECM‐related genes (COL1A1, COL1A2 and α‐SAM), tumorigenesis (Afp, Gpc3 and Ly6d) and inflammation (TNF‐α, IL‐6 and IL‐β) (Figure S1F–H, Supporting Information). Immunohistochemical (IHC) staining and Western blot analysis showed that CCl_4_ treatment increased the FGF9 level in the livers of mice, especially those in the HFHC diet and CCl_4_‐treated groups (Figure 1E,F). Thus, these results indicate that both HFHC diets feeding and CCl_4_ might be a potent stimulus for increasing FGF9 expression, and CCl_4_ and HFHC diets could show synergistic effects to worsen pathophysiology of NASH.

### 2.2 FGF9 Promotes HCC Cell Proliferation, Migration and Invasion and Induces the Expression of ECM‐Related Genes

**Figure 2:**
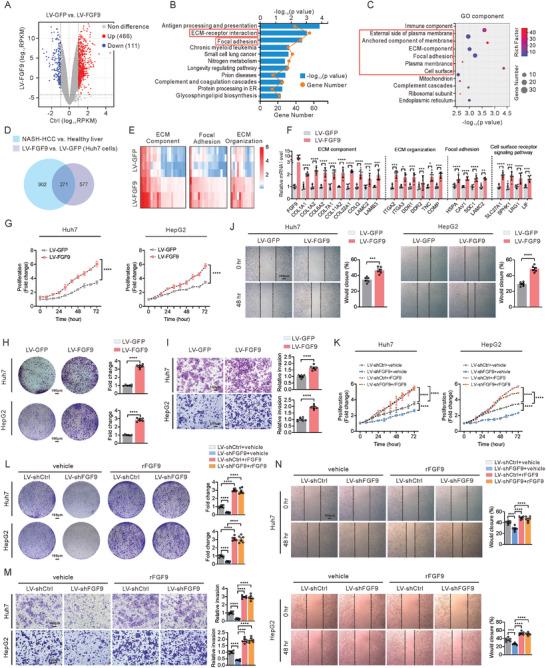
Overexpression of FGF9 induces ECM‐related genes expression and promotes HCC cell proliferation, migration and invasion. A) Volcano plot showing the DEGs in Huh7 cells infected with LV‐GFP or LV‐FGF9. B) KEGG pathway enrichment analysis of upregulated genes in LV‐FGF9‐infected Huh7 cells. The orange dots represent the number of genes in each annotated pathway, and the length of each column is based on the p value. C) GO enrichment analysis of cellular component categories for upregulated genes in LV‐FGF9‐infected Huh7 cells. The sizes of the dots represent the numbers of genes in the gene sets, and the color represents the rich factor of the enrichment. D) Venn diagram showing the overlapping significantly differentially expressed transcripts in NASH‐driven HCC identified in mice treated with CCl_4_ and the Western diet (GSE95140, *p*<0.01) and in Huh7 cells infected with LV‐FGF9 (*p*<0.01). E and F) Heatmap of transcriptome data (E) and RT‐qPCR data (F) showing the mRNA levels of genes involved in the “ECM component”, “ECM organization”, “Focal adhesion” and “Cell surface receptor signaling pathway” pathways. G–J) Cell proliferation assays (G), colony formation assays (H), invasion assays (I) and migration assays (J) of Huh7 and HepG2 cells infected with LV‐GFP or LV‐FGF9. K–N) Cell proliferation assays (K), colony formation assays (L), invasion assays (M) and migration assays (N) of FGF9 knockdown‐Huh7 and HepG2 cells infected with or without rFGF9 treatment. The data are shown as the means ± SEM. *p < 0.05, **p < 0.01, ***p < 0.005, ****p < 0.001; 2‐way ANOVA (G, H, I, J, K, L, M, N), 2‐tailed Student's *t* test (F).

To determine the function of FGF9 on the tumorigenesis, we first performed RNA‐seq analysis on Huh7 cells overexpressing FGF9. Preliminary analysis of the RNA‐seq data showed that 466 genes were upregulated, and 111 genes were downregulated (**Figure** **2**A). Kyoto Encyclopedia of Genes and Genomes (KEGG) pathway analysis indicated that the upregulated genes were significantly enriched in functionally annotated ECM‐related pathways, including “ECM‐receptor interaction” and “Focal adhesion”, which were associated with NASH progress and promoted HCC (Figure 2B). In line with the KEGG analysis, Gene Ontology (GO) analysis of cellular components showed 6 categories, which were linked to extracellular component, were positively correlated with FGF9, suggesting that these changes could be driven by ECM‐dependent mechanisms (Figure 2C).

To further explore the correlation between the differentially expressed genes (DEGs) related to FGF9 overexpression and NASH, we overlapped our transcriptome data with previously reported hepatic transcriptome data from the mouse model of NASH‐driven HCC.^[^ ^28^ ^]^ A total of 902 and 577 DEGs were identified in mice with NASH‐driven HCC (GSE95140, *p*<0.01) and FGF9‐overexpressing Huh7 cells (*p*<0.01), respectively, compared to their respective controls. Additionally, 271 DEGs overlapped between the two datasets (Figure 2D). The heatmap and the real‐time quantitative PCR (RT‐qPCR) results confirmed that FGF9 overexpression increased the expression of genes from the overlapping DEGs, involved in ECM signaling pathways, including “ECM component”, “ECM organization”, “Focal adhesion” and “Cell surface receptor signaling pathway” (Figure 2E,F).

Next, we investigated the effect of FGF9 on tumorigenesis in the Huh7 and HepG2 cell lines. The MTT and colony formation assays showed that overexpression of FGF9 by lentiviral (LV‐FGF9) transfection significantly promoted Huh7 and HepG2 cells proliferation compared with that of the control cells (LV‐GFP transfected) (Figure 2G,H). Moreover, Transwell assay also demonstrated that FGF9 overexpression significantly increased the invasion of Huh7 and HepG2 cells (Figure 2I). The wound healing assay further showed that FGF9 overexpression promoted Huh7 and HepG2 cells migration (Figure 2J). Moreover, knockdown of FGF9 exhibited decrease in Huh7 and HepG2 cells proliferation, as assessed by MTT and colony formation assays, however, cells proliferation was increased in the presence of exogenously added recombinant human FGF9 (rFGF9) protein in both Huh7 and HepG2 cells, even with FGF9 knockdown (Figure 2K,L). Concomitantly, FGF9 knockdown diminished the invasion and migration of Huh7 and HepG2 cells, and rFGF9 treatment restored the tumorigenic activity of FGF9 knockdown Huh7 and HepG2 cells (Figure 2M,N).

Due to the involvement of hepatic stellate cells (HSCs) in liver fibrosis. Therefore, we investigated whether pro‐fibrotic action of FGF9 may be due to its direct effect on HSCs, a main fibrogenic effector cell in the liver, we freshly isolated HSCs and incubated them with increasing concentrations of FGF9 (20, 40, and 60 nM) in vitro. Our results showed that primary HSCs failed to respond to rFGF9 in vitro, both at baseline and upon activation by transforming growth factor (TGF‐β, a potent HSCs activator) as measured by cell proliferation, migration and invasion (Figure S2, Supporting Information).

Together, these findings indicate that exposure to exogenous FGF9 directly triggered tumorigenic activation in vitro, accompanying ECM as the main active ingredient, as evidenced by characteristic changes in the expression ECM‐related genes.

### 2.3 FGF9 Increased the Expression of Genes Related to the ECM in a β‐Catenin‐Dependent Manner

**Figure 3:**
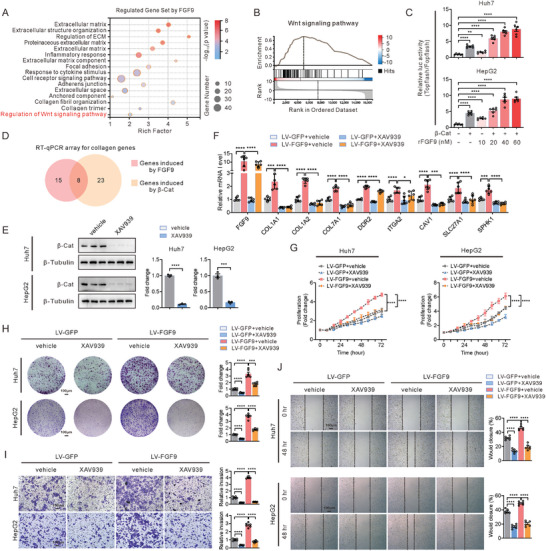
FGF9 regulates the expression of ECM‐related genes and HCC cells proliferation, migration and invasion in a β‐catenin‐dependent manner. A) Bubble chart showing the results of GSEA based on GO cellular component categories for the overlapping DEGs from LV‐FGF9‐infected Huh7 cells and liver tissue from the NASH‐driven HCC model (GSE95140, *p*<0.01). The sizes of the dots represent the numbers of genes in the gene sets, and the transparency represents the FDR of the enrichment, FDR, false discovery rate. B) GSEA revealed that the DEGs were enriched in “Wnt signaling pathway”. NES, normalized enrichment score; FDR, false discovery rate. C) Analysis of changes in TOP‐Flash reporter activity induced in Huh7 and HepG2 cells by treatment with serially increasing concentrations of rFGF9. D) Venn diagram showing 8 significant DEGs (related to the ECM) induced by β‐catenin and FGF9 in Huh7 cells. E) Western blots analysis of β‐catenin in Huh7 and HepG2 cells with or without XAV‐939 treatment. F) RT‐qPCR analysis of the genes (from the Venn diagram result of D) in Huh7 cells infected with LV‐GFP or LV‐FGF9 and treated with or without XAV‐939. G–J) Cell proliferation assays (G), colony formation assays (H), invasion assays (I) and migration assays (J) of Huh7 and HepG2 cells infected with LV‐GFP or LV‐FGF9 and treated with or without XAV‐939. The data are shown as the means ± SEM. *p < 0.05, **p < 0.01, ***p < 0.005, ****p < 0.001; 1‐way ANOVA (C), 2‐tailed Student's *t* test (F), 2‐way ANOVA (F, G, H, I, J).

To gain insight into the underlying mechanisms by which FGF9 regulated ECM, we analyzed the overlapping DEGs identified in FGF9‐overexpressing Huh7 cells and the NASH‐driven HCC model by GO enrichment analysis. Notably, the results showed that, except for the top‐ranked enriched pathways positively correlated with FGF9 were related to ECM function, DEGs were also involved in the term “Regulation of Wnt signaling pathway”, suggesting that the expression of these DEGs may be driven by β‐catenin‐dependent mechanisms (**Figure** **3**A). Additionally, gene set enrichment analysis (GSEA) of cellular components showed that Wnt signaling pathway was positively correlated with FGF9 expression (Figure 3B). Thus, using Wnt pathway‐specific luciferase reporters, we further explored whether FGF9 regulates the Wnt pathway. The results identified that the fluorescence intensity of TOP‐Flash, which was activated by β‐catenin, was significantly enhanced by treatment with increasing amounts of rFGF9, indicating a dose‐dependent effect of rFGF9 on the transcriptional activation of β‐catenin (Figure 3C).

Since FGF9 induced the transcriptional activity of β‐catenin, which plays an important role in ECM production,^[^ ^13^ ^]^ we hypothesized that the main signaling pathway by which FGF9 regulates ECM production and subsequent fibrosis in the liver is partially stimulated through activation of β‐catenin. To determine whether the FGF9‐mediated transcription of genes related to the ECM, we screened the partial ECM pathway using RT‐qPCR in Huh7 cells transfected with FGF9 or β‐catenin expression vectors. We confirmed that 23 genes related to the ECM were induced by FGF9 and that 15 genes related to ECM were induced by β‐catenin and found that diverse ECM‐related genes were equally regulated by FGF9 and β‐catenin in Huh7 cells (Figure 3D). Due to the pharmacologic inhibition of β‐catenin signaling by XAV‐939 stabilizes the β‐catenin destruction complex, decreased cytoplasmic retention and nuclear import of β‐catenin, and efficiently inhibited β‐catenin‐targeted gene transcription.^[^ ^29^ ^]^ XAV‐939 was introduced to further determine whether β‐catenin is necessary for the FGF9‐mediated induction of ECM‐related genes. We first confirmed that XAV‐939 almost blocked the β‐catenin expression (Figure 3E). Then, we found that XAV‐939 treatment almost completely abolished FGF9‐induced activation of TOP‐Flash and ECM‐related gene expression compared to that in the control groups (Figure 3F). Consistent with the gene expression patterns, FGF9 markedly promoted the proliferation, migration and invasion of Huh7 and HepG2 cells; however, XAV‐939 treatment almost blocked the action of FGF9 (Figure 3G–J).

### 2.4 FGF9 Regulates the Stability of β‐Catenin

**Figure 4:**
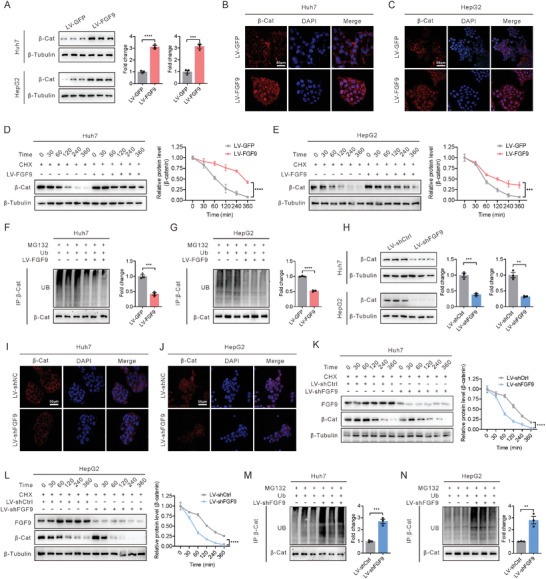
FGF9 targets and regulates β‐catenin stability in HCC cells. A) Upregulation of endogenous β‐catenin in Huh7 and HepG2 cells infected with LV‐GFP or LV‐FGF9, as examined by Western blot analysis. B and C) Upregulation of endogenous β‐catenin in Huh7 (B) and HepG2 (C) cells infected with LV‐GFP or LV‐FGF9, as examined by confocal microscopy. D and E) Comparison of β‐catenin degradation in Huh7 (D) and HepG2 (E) cells infected with LV‐GFP or LV‐FGF9 and then treated with CHX in a time series. F and G) Ubiquitination of β‐catenin in Huh7 (F) and HepG2 (G) cells in response to infection of LV‐FGF9. H) Down‐regulation of endogenous β‐catenin in Huh7 and HepG2 cells infected with LV‐shCtrl or LV‐shFGF9, as examined by Western blot analysis. I and J) Down‐regulation of endogenous β‐catenin in Huh7 (I) and HepG2 (J) cells infected with LV‐shCtrl or LV‐shFGF9, as examined by confocal microscopy. K and L) Comparison of β‐catenin degradation in Huh7 (K) and HepG2 (L) cells infected with LV‐shCtrl or LV‐shFGF9 and then treated with CHX in a time series. M and N) Ubiquitination of β‐catenin in Huh7 (M) and HepG2 (N) cells in response to infection of LV‐shFGF9. The data are shown as the means ± SEM. *p < 0.05, **p < 0.01, ***p < 0.005, ****p < 0.001; 2‐tailed Student's t test (A, F, G, H, M, N), 2‐way ANOVA D, E, K, L).

Next, we further examined the internal mechanism by which FGF9 regulates β‐catenin. First, we demonstrated that forced expression of FGF9 significantly increased the levels of endogenous and exogenous β‐catenin proteins in Huh7 and HepG2 cells by western blot (**Figure** **4**A; Figure S3A, Supporting Information). In addition, the increased nuclear accumulation of both endogenous and exogenous β‐catenin was detected after FGF9 overexpression in Huh7 and HepG2 cells by immunofluorescence analysis (Figure 4B,C; Figure S3B, Supporting Information). However, the mRNA levels of β‐catenin were not changed (Figure S3C, Supporting Information). In addition, time‐course treatment of Huh7 and HepG2 cells with cycloheximide (CHX) showed degradation of β‐catenin with prolonged treatment time, and this degradation was attenuated after FGF9 overexpression in liver cancer cells (Figure 4D,E). Next, we found that the differences of stabilization and nuclear localization of the β‐catenin protein by FGF9 were diminished after MG132 treatment (Figure S3D,E, Supporting Information). In addition, FGF9 overexpression significantly attenuated the ubiquitination of β‐catenin (Figure 4F,G), suggesting that the events induced by FGF9 were dependent on the ubiquitin‐proteasome‐mediated protein degradation pathway. Then, we further evaluated the change in β‐catenin stability in response to knockdown of endogenous FGF9 in liver cancer cells. Knockdown of FGF9 resulted in diminishing β‐catenin protein level in Huh7 and HepG2 cells (Figure 4H–J). In addition, FGF9 knockdown Huh7 and HepG2 cells exhibited increased degradation of β‐catenin with prolonged CHX treatment (Figure 4K,L). Similarly, its mRNA level did not change (Figure S3F, Supporting Information). In addition, knockdown of FGF9 markedly increased the ubiquitination of β‐catenin in Huh7 and HepG2 cells (Figure 4M,N). However, inhibiting FGF9 expression had no significant effect on the protein stability of β‐catenin in MG132‐treated cells (Figure S3G,H, Supporting Information). Together, these results indicate that FGF9 is involved in regulating β‐catenin stability via the ubiquitin‐proteasome pathway.

### 2.5 FGF9 Promotes the Release of β‐Catenin from the Destruction Complex via ERK1/2‐GSK‐3β

**Figure 5:**
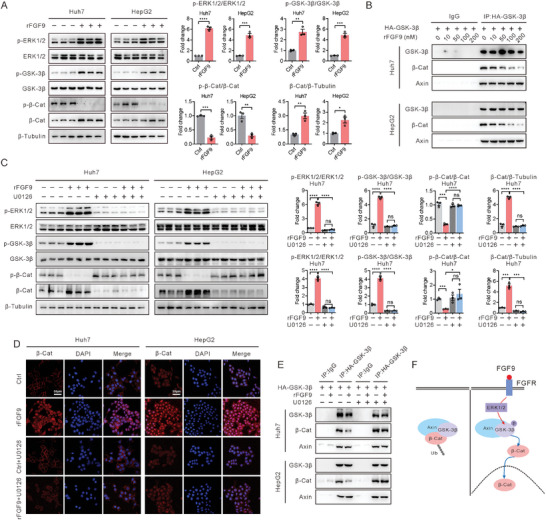
FGF9 regulates the stability of β‐catenin via the ERK1/2‐GSK‐3β pathways in Huh7 and HepG2 cells. A) Representative Western blot analysis of p‐ERK1/2, p‐GSK‐3β, and p‐β‐catenin in Huh7 and HepG2 cells 15 min after rFGF9 treatment and quantification of the target protein bands using ImageJ software (*n* = 3). B) Interaction of endogenous β‐catenin with GSK‐3β in Huh7 and HepG2 cells transfected with HA‐GSK‐3β. C) Huh7 and HepG2 cells were pretreated with or without U0126 (10 mM) for 1 h and were then treated with rFGF9 (200 nM) or PBS for 15 min. Western blot analysis of p‐ERK1/2, p‐GSK‐3β, and p‐β‐catenin in Huh7 and HepG2 cells, and quantification of the target protein bands using ImageJ software (n = 3). D) Confocal microscopy analysis of β‐catenin in Huh7 and HepG2 cells. E) Interaction of endogenous β‐catenin with GSK‐3β in Huh7 and HepG2 cells transfected with HA‐GSK‐3β. F) Schematic representation of the proposed mechanism by which FGF9 regulates the stability of the β‐catenin protein. The data are shown as the means ± SEM. ***p < 0.005, ****p < 0.001; 2‐tailed Student's *t* test (A), 2‐way ANOVA (C).

The above results raise questions about the mechanism by which FGF9 affects β‐catenin stability. The destruction complex is at the center of Wnt pathway regulation through inactivation of GSK‐3β signaling to release β‐catenin from the destruction complex. This relationship prompted us to investigate the effects of FGF9 on the regulation of the interaction between β‐catenin and the destruction complex. First, we explored whether the increase in the content of endogenous β‐catenin induced by rFGF9 treatment is dependent on decreased phosphorylation of β‐catenin through the regulation of GSK‐3β activity. As expected, rFGF9 treatment significantly increased the phosphorylated GSK‐3β levels, in parallel with the decreased phosphorylation levels of β‐catenin. Moreover, ERK1/2 phosphorylation levels, which was the downstream of FGF9, were also increase in Huh7 and HepG2 cells after rFGF9 treatment (**Figure** **5**A). Besides, the amount of β‐catenin in the immunocomplexes was dose‐dependently decreased in response to rFGF9 treatment, as determined by exogenous HA‐GSK‐3β‐specific immunoprecipitation assays. However, the abundance of Axin, another key component of the destruction complex, was rarely changed in the immunoprecipitated fraction in response to rFGF9 treatment (Figure 5B).

To further delineate the signaling pathway by which FGF9 stimulates cellular activation of β‐catenin, U0126, a specific ERK1/2 inhibitor, was used to treat Huh7 and HepG2 cells. Western blot analysis indicated that rFGF9 treatment increased the phosphorylation of ERK1/2 and GSK‐3β, accompanied by decreased phosphorylation of β‐catenin, while cotreatment with U0126 and rFGF9 reversed the phosphorylation status of ERK1/2, GSK‐3β and β‐catenin in Huh7 and HepG2 cells (Figure 5C). Immunofluorescence analysis further confirmed that U0126 inhibited the increase of rFGF9 induced β‐catenin content (Figure 5D). Moreover, the interaction between β‐catenin and GSK‐3β was examined in rFGF9‐treated Huh7 and HepG2 cells with or without U0126 cotreatment. In contrast to rFGF9 treatment alone, U0126 cotreatment almost completely abolished the effects of rFGF9 on the interaction between β‐catenin and GSK‐3β. Consistent with the increase in the phosphorylated ERK1/2 level induced by U0126 treatment, the content of β‐catenin in the immunoprecipitated fraction was rarely changed in response to rFGF9 treatment (Figure 5E), suggesting that FGF9 positively regulates β‐catenin activity through ERK1/2 and GSK‐3β phosphorylation (Figure 5F).

Treatment with rFGF9 alone increased the TOP‐Flash activity and the expression of ECM‐related genes; however, cotreatment with U0126 almost completely blocked these effects of rFGF9 on β‐catenin activity (Figure S4A,B, Supporting Information). In addition, functional studies showed that U0126 treatment effectively inhibited cell proliferation, migration and invasion of rFGF9‐treated Huh7 and HepG2 cells (Figure S4C–S4F, Supporting Information). These results further indicate that FGF9 exerts its effects in an ERK1/2‐GSK‐3β pathway‐dependent manner.

### 2.6 FGF9 Deletion in Hepatocytes Attenuates NASH‐Driven HCC

**Figure 6:**
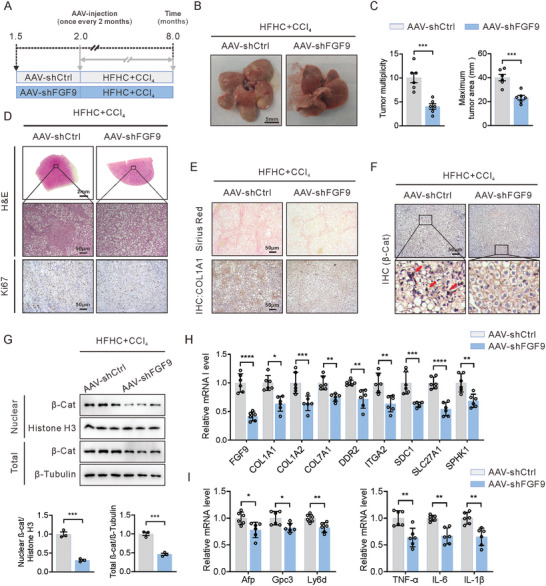
Hepatocyte‐specific FGF9 knockdown attenuates NASH‐driven HCC in HFHC diets‐fed, CCl^4^‐treated wild‐type mice. A) Schematic illustration of the experimental design: Mice were injected with AAV‐shFGF9 or AAV‐shCtrl via the tail vein for every 2 months. After the first injection, the mice were fed a HFHC diet and treated with CCl_4_ for 6 months. B and C) Representative macroscopic images (B) and quantification of the tumor burden and maximal area (C) in AAV‐shFGF9‐ or AAV‐shCtrl‐injected mice fed a HFHC diet and treated with CCl_4_ for 6 months. D) Representative H&E staining and Ki67 staining (IHC) in liver sections from AAV‐shFGF9‐ or AAV‐shCtrl‐injected mice fed a HFHC diet and treated with CCl4 for 6 months. E) Representative histological staining of collagen fibers (Sirius red) and COL1A1 (IHC) in liver sections from AAV‐shFGF9‐ or AAV‐shCtrl‐injected mice fed a HFHC diet and treated with CCl_4_ for 6 months. F) Representative histological staining of β‐catenin (IHC). G) Total and nuclear β‐catenin level, as determined by Western blot analysis. H and I) RT‐qPCR analysis of the mRNA expression of ECM‐related genes (H), tumor markers and inflammation‐related genes (I) in whole liver tissue from AAV‐shFGF9‐ or AAV‐shCtrl‐injected mice fed a HFHC diet and treated with CCl_4_ for 6 months. The data are shown as the means ± SEM. *p < 0.05, **p < 0.01, ***p < 0.005, ****p < 0.001; 2‐tailed Student's *t* test (C, E, I).

To address the contribution of FGF9 in NASH‐driven HCC, FGF9 was specifically knocked down in the liver by infection with an adeno‐associated virus (AAV‐shFGF9), and the susceptibility to HCC was examined in the model of spontaneous NASH‐driven HCC, in which mice were treated with a HFHC diet and CCl_4_ after FGF9 knockdown. Mice were first injected with AAV (AAV‐shFGF9 or AAV‐shCtrl), and then re‐administration of AAV 3 months after the initial AAV‐injection to enable sustained knockdown of FGF9 in the liver. The AAV‐shFGF9 injected mice exhibited profound depletion of FGF9 in liver extracts compared to AAV‐shCtrl‐injected mice (Figure S5, Supporting Information). Then, AAV‐injected mice were fed the HFHC diet in combination with a low dose of CCl_4_ treatment for 6 months (**Figure** **6**A). Tumorigenesis occurred in AAV‐shCtrl injected mice after HFHC and CCl_4_ treatment, manifested as an increased tumor number and tumor area, whereas injection of AAV‐shFGF9 did not lead to tumorigenesis in mice after the same stimulation (Figure 6B,C). Moreover, hematoxylin‐eosin (H&E) and Ki67 staining showed less hepatic tumor formation and proliferation activity in AAV‐shFGF9‐injected mice than in AAV‐shCtrl‐injected mice (Figure 6D). In addition, Sirius red staining of liver sections from AAV‐shFGF9‐injected mice revealed decreased hepatic fibrosis compared with that in the control groups. These changes were accompanied by decreased expression of COL1A1, an important ECM component in HCC development, in the livers of AAV‐shFGF9‐injected mice, as shown by IHC staining (Figure 6E).

Next, we sought to address the expression level and subcellular localization of β‐catenin in AAV‐shCtrl and AAV‐shFGF9 HFHC diet and CCl_4_‐treated mice by immunohistochemistry. Importantly, it is only in the livers of HFHC diet and CCl_4_‐treated mice that β‐catenin was present in both the nucleus and membrane, consistent with the activation of Wnt signaling reported in the late stage of human HCC. However, β‐catenin was lower and localized predominantly at the plasma membrane in hepatocytes from AAV‐shFGF9‐injected mice (Figure 6F). In addition, Western blot analysis confirmed that the levels of both total and nuclear β‐catenin were decreased in livers from AAV‐shFGF9‐injected mice (Figure 6G).

Furthermore, deletion of FGF9 decreased the mRNA expression of EMC, hepatic tumor markers (such as Afp, Gpc3 and Ly6d) and inflammation‐related factors (TNF‐α, IL‐6 and IL‐1β) in the livers of HFHC diet and CCl_4_‐treated mice (Figure 6H,I).

### 2.7 FGF9 Promotes Hepatic ECM Accumulation and HCC in Mice Fed a HFHC Diet

**Figure 7:**
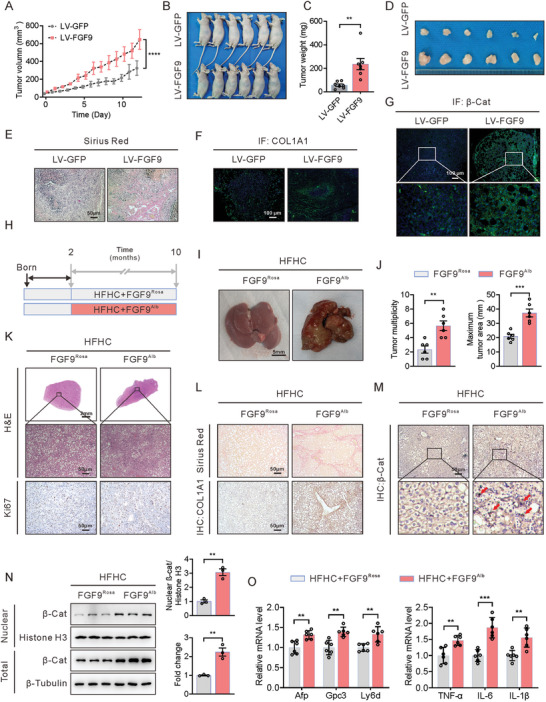
Hepatocyte‐specific FGF9 overexpression exacerbates xenograft growth in nude mice and the development of HFHC diet‐driven HCC tumors in FGF9 transgenic mice. A–D) Control and FGF9‐overexpressing Huh7 cells were subcutaneously injected into BALB/c nude mice to observe tumor growth, tumor volumes and weights. E–G) Representative histological staining of collagen fibers (E), COL1A1 (F) and β‐catenin (G) in tumor nodules. H) Schematic illustration of the experimental design, with induction of tumorigenesis in the livers of FGF9^Rosa^ and FGF9^Alb^ mice fed a HFHC diet for 8 months. I and J) Macroscopic images of livers from FGF9^Rosa^ and FGF9^Alb^ mice fed a HFHC diet for 8 months (I), with quantification of the tumor burden and maximal area (J). K) Representative H&E staining and Ki67 staining (IHC) in liver sections from FGF9^Rosa^ and FGF9^Alb^ mice. L) Representative histological staining of collagen fibers (Sirius red) and COL1A1 (IHC) in liver sections from FGF9^Rosa^ and FGF9^Alb^ mice. M) Representative histological staining of β‐catenin (IHC) in liver sections from FGF9^Rosa^ and FGF9^Alb^ mice. N) Nuclear β‐catenin level, as determined by Western blot analysis. O) RT‐qPCR analysis of the mRNA expression of tumor markers and inflammation‐related genes in whole liver tissue from FGF9^Rosa^ and FGF9^Alb^ mice. The data are shown as the means ± SEM. **p < 0.01, ***p < 0.005, ****p < 0.001; 2‐tailed Student's *t* test (C, J, N, O), 2‐way ANOVA (A).

To further explore the role of FGF9 in the induction of fibrosis and NASH‐driven HCC, a subcutaneous xenograft model in nude mice and a mouse model of HFHC diet‐induced NASH were introduced. Subcutaneous xenograft tumors with FGF9 overexpression exhibited a more obvious increase in tumor growth than those derived from cells expressing the control vector, as seen by the higher tumor volume and weight (**Figure** **7**A–D). In addition, overexpression of FGF9 resulted in severe fibrosis in subcutaneous tumors compared to that in the control group (Figure 7E). Immunofluorescence staining showed that overexpression of FGF9 increased the accumulation of COL1A1 in subcutaneous tumors compared with that in the control group (Figure 7F). Moreover, the both total and nuclear β‐catenin protein levels were significantly higher in the FGF9‐overexpressing group than in the control group, and β‐catenin was almost completely localized at the membrane of tumor cells in the control group (Figure 7G). Thus, the subcutaneous xenograft assay results further confirmed that FGF9 is required for fibrosis and liver cancer cells growth in vivo.

To further explore the contribution of FGF9 to NASH‐driven HCC, liver‐specific FGF9 transgenic (FGF9^Alb^) mice were generated by crossing FGF9 Rose26 mice (FGF9^Rosa26^) with mice with albumin promoter‐induced Cre expression (Alb‐Cre mice), which resulted in a 6‐fold increase in liver FGF9 expression (both mRNA and protein) compared with that in control mice (FGF9^Rosa26^ mice) (Figure S6, Supporting Information). To examine the impact of FGF9 on NASH‐driven hepatic tumorigenesis, we fed FGF9^Alb^ mice a HFHC diet for 10 months (Figure 7H). Like HFHC diet and CCl_4_‐treated mice, FGF9^Alb^ mice fed the HFHC diet exhibited visible spontaneous tumors in the liver. Consistent with this observation, FGF9^Alb^ mice developed marked hepatic tumor formation and proliferation activity compared with control mice (Figure 7I–K). In addition, FGF9^Alb^ mice exhibited increased fibrosis and COL1A1 content, along with increased mRNA expression of ECM‐related genes (Figure 7L; Figure S7, Supporting Information).

In addition, in contrast to the localization of β‐catenin in the control livers, i.e., predominantly at the plasma membrane, the levels of both nuclear and membrane β‐catenin were increased in livers from FGF9^Alb^ mice, as shown by IHC and Western blot analyses (Figure 7M,N). Moreover, the expression of HCC markers, including Afp, Gpc3 and Ly6d, in livers was higher in the FGF9^Alb^ mice, and this outcome was accompanied by enhanced expression of inflammation‐related genes (TNF‐α, IL‐6 and IL‐1β) (Figure 7O). These results indicate that the phenotype and β‐catenin localization pattern reportedly observed in human HCC were largely reproduced in FGF9^Alb^ mice fed the HFHC diet.

FGF9^Alb^ mice had an increased liver/body weight ratio, a modest increase in hepatocyte proliferation and increased β‐catenin level after being fed SD (Figure S8, Supporting Information); however, these mice did not develop hepatic tumors at 10 months of age. These findings suggest that HFHC diet consumption might be necessary for promoting HCC development in FGF9 ^Alb^ mice.

### 2.8 Suppression of β‐Catenin Abrogates FGF9‐Driven ECM Accumulation and HCC

**Figure 8:**
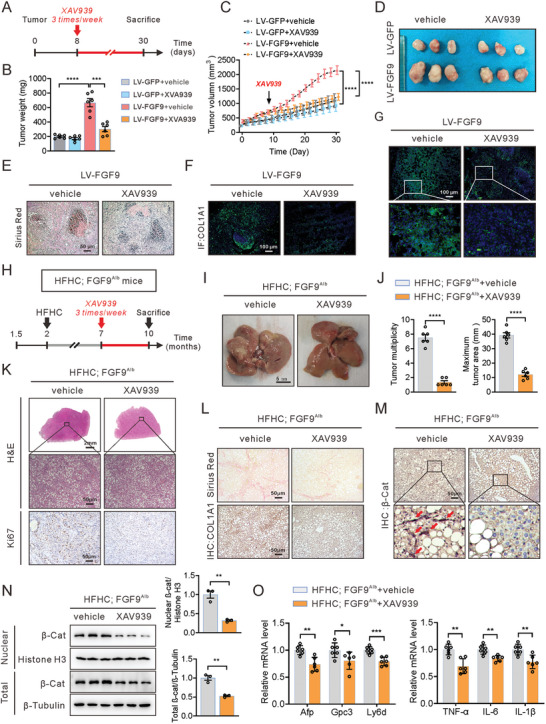
Blockade of the Wnt/β‐catenin pathway reverses the growth of xenografts in nude mice and development of HFHC diet‐driven HCC in FGF9 transgenic mice. A) Schematic illustration of the experimental design for establishment of the xenograft model followed by XAV‐939 treatment. FGF9‐overexpressing Huh7 cells were subcutaneously injected into BALB/c nude mice, and the mice were treated with or without XAV‐939. B–D) Tumor volumes (B), macroscopic images of subcutaneous tumors (C) and volumes (D) in nude mice. E–G) Representative histological staining of collagen fibers (E), COL1A1 (F) and β‐catenin (G) in tumor nodules. H) Schematic illustration of the experimental design, with induction of tumorigenesis in the livers of FGF9^Alb^ mice fed a HFHC diet and then treated with XAV‐939 (3 months). I and J) Macroscopic images of livers from HFHC diet‐fed FGF9^Alb^ mice treated with or without XAV‐939 (I) and quantification of the tumor burden and maximal area (J). K–M) Representative H&E staining and staining of Ki67 (K), collagen fibers (L), COL1A1 (L) and β‐catenin (M) in liver sections of FGF9^Alb^ mice treated with or without XAV‐939. N) Nuclear β‐catenin level, as determined by Western blot analysis. O) mRNA levels of tumor marker and inflammation‐related genes in whole liver tissue. The data are shown as the means ± SEM. *p < 0.05, **p < 0.01, ***p < 0.005, ****p < 0.001; 2‐tailed Student's *t* test (J, O), 2‐way ANOVA (B, C).

XAV‐939 treatment inhibited FGF9‐mediated β‐catenin signaling in Huh7 and HepG2 cells, thereby implying a strong correlation between FGF9 function and β‐catenin‐mediated NASH‐driven HCC. Thus, XAV‐939 (3 times/week) was introduced to examine the efficacy of FGF9 in the stimulation of hepatic tumor initiation and progression. Consistent with our hypothesis, the subcutaneous xenograft tumors with FGF9 overexpression in nude mice exhibited an aggressive growth pattern of tumor nodules, and these changes were significantly suppressed upon XAV‐939 administration, as shown by the decreased tumor volume and weight (**Figure** **8**A–D). In line with these results, XAV‐939 treatment ameliorated fibrosis in tumor nodules (Figure 8E), in agreement with the lower expression of COL1A1 (Figure 8F). Moreover, immunofluorescence analysis confirmed that β‐catenin protein expression was decreased by XAV‐939 treatment, and β‐catenin was almost completely localized at the membrane of tumor cells (Figure 8G).

Additionally, consistent with findings in the nude mouse model treated with XAV‐939, spontaneous development of HCC in FGF9^Alb^ mice fed a HFHC diet was detected, while the formation of HCC lesions, in terms of both the nodule number and nodule size, was significantly inhibited when the animals were treated with XAV‐939 (Figure 8H–K). Moreover, Sirius Red staining showed that treatment of XAV‐939 resulted in a notable decrease in liver fibrosis in FGF9^Alb^ mice fed a HFHC diet. We further confirmed the alteration of COL1A1 content by histopathological analysis of COL1A1 staining, which was significantly reduced in livers from FGF9^Alb^ mice after XAV‐939 treatment (Figure 8L). We also observed that the hepatic levels of both nuclear and total β‐catenin were decreased in FGF9^Alb^ mice (Figure 8M,N), consistent with the attenuated expression of ECM related genes, Afp, Gpc3 and Ly6d, markers of tumorigenesis, and the decreased expression of genes associated with inflammation (Figure 8O; Figure S9, Supporting Information). Notably, measurement of the liver triglyceride, food intake, body weight and organ/body weight indicated that XAV‐939 treatment had minimal toxicity for mice (Figure S10, Supporting Information). Collectively, these data indicate that FGF9 is indispensable for the modulation of ECM synthesis and organization and promotes liver fibrosis accompanied by NASH‐driven HCC.

## 3 Discussion

Although FGF9 has already been reported to enhance the proliferation, invasion and migration of HCC cells in vitro,^[^ ^30^, ^31^, ^32^, ^33^, ^34^ ^]^ however, the role of FGF9 in liver tumor formation in vivo, especially in NASH‐driven HCC, is still fully understood.

NASH‐driven HCC development is a growing public health burden that is expected to increase globally because of its association with obesity and type 2 diabetes.^[^ ^1^ ^]^ Accumulating evidence has demonstrated that ECM accumulation, which is the most important characteristic of NASH, plays a critical role in the process of HCC.^[^ ^6^, ^9^ ^]^ Similar with the observations in several reported NASH‐driven HCC models which exhibited increased ECM content,^[^ ^23^, ^28^, ^35^, ^36^ ^]^ accumulation of ECM has been found in clinical patients and laboratory animal models of NASH‐driven HCC in our study, along with the increased hepatic FGF9 expression observed. In recent years, researchers have increasingly recognized the important role of FGF9 in the development of several types of cancers, such as breast cancer, ovarian cancer, gastric cancer and colon cancer,^[^ ^33^, ^37^, ^38^, ^39^ ^]^ however, it is still obscure whether FGF9 participates the NASH driven HCC. Herein, we confirmed that the FGF9 controlled hepatic ECM accumulation in the NASH‐driven HCC model and established that FGF9 stimulates liver fibrosis and promotes the occurrence of HCC via ECM synthesis.

The ECM not only is an important feature of NASH but also promotes HCC progression through complex signaling pathways. ECM has unique biochemical and biomechanical properties that contribute to the growth, survival, adhesion and invasion of tumor cells.^[^ ^40^ ^]^ In addition to providing mechanical strength, ECM components can also regulate multiple signaling cascades through their ability to bind to specific receptors (such as integrins) and growth factors, which thereby regulates their distribution, activation, and presentation. Surrounding cells sense changes in the ECM via specific transmembrane receptors, which in turn regulate specific signaling pathways within the cell in response to external stimuli. Aberrant ECM accumulation plays key roles in regulating cancer development. Increased levels of ECM components, such as collagens, laminins, fibronectin, proteoglycans and glycosaminoglycans, lead to phenotypic and behavioral changes in the liver.^[^ ^41^ ^]^

In the present study, we established a NASH‐driven HCC mice model and confirmed that an HFHC diet and along with low‐dose CCl_4_ treatment could promote HCC approach. In addition, we confirmed that increased FGF9 promoted the tumor formation in another animal model, in which the liver‐specific FGF9 transgenic mice feeding with HFHC diet only without CCl_4_ treatment. The histological examination of FGF9 transgenic mice feeding with HFHC diet exhibited hepatic steatosis, inflammation and ballooning, in especial ECM accumulation were like those in the model of NASH induced by HFHC diet feeding and CCl_4_ treatment, indicating that the up‐regulation of hepatic FGF9 may be the cause of ECM accumulation and NASH‐driven HCC.

Furthermore, transcriptome analysis showed that ECM homeostasis‐related pathways, including “ECM‐receptor interaction” and “Focal adhesion,” were significantly enrichment in Huh7 cells overexpressing FGF9 and closely replicated the reported transcriptomic hallmarks of NASH‐driven HCC induced by diethylnitrosamine (DEN) treatment and western diet feeding.^[^ ^28^, ^36^ ^]^ In addition, in vivo studies confirmed increased ECM‐related gene expression in liver tumors of HFHC diet‐fed FGF9 TG mice. Consistent with the gene expression patterns, both in vitro and in vivo experiments showed that increasing or decreasing the expression of FGF9 in the mouse liver led to significant increases or reductions, respectively, in ECM synthesis and subsequent fibrosis.

Mechanistically, our results provide a plausible explanation for the mechanism by which FGF9 stabilizes β‐catenin to facilitate ECM accumulation and NASH‐driven HCC. Multiple studies reported that FGF9 signaling pathway interacts with Wnt signaling pathway and participates in various physiological and pathological processes. For example, FGF9 promotes HAS2 expression via Wnt/β‐catenin/TCF7L2 pathway.^[^ ^42^ ^]^ FGF9 promotes cisplatin resistance via decreased APC expression and activation of the Wnt/β‐catenin signaling pathway in colorectal cancer cells.^[^ ^43^ ^]^ In addition, FGF9, which is considered as the downstream target gene of Wnt/β‐catenin signaling, have been reported to participate the wound repair^[^ ^44^ ^]^ and ovarian endometrioid adenocarcinomas.^[^ ^45^ ^]^ However, these studies did not delve into the mechanism by which FGF9 regulates the Wnt/β‐catenin signaling pathway. We found that changes in β‐catenin expression in samples of NASH‐driven HCC were positively correlated with FGF9 expression; in addition, our results showed that FGF9 promotes increased β‐catenin stability in NASH‐driven HCC by regulating the ERK1/2‐GSK‐3β signaling pathway. Given that the increase in FGF9 reduced the interaction between β‐catenin and GSK‐3β, FGF9 may act as a novel stimulus to inhibit the GSK‐3β‐mediated β‐catenin ubiquitination, subsequently activating the transcriptional activity of β‐catenin. Thus, the stabilized β‐catenin protein translocated to the nucleus and subsequently upregulates its downstream targets, which facilitates ECM accumulation and HCC progression, which will help us further understand the roles of FGF9 in NASH‐driven HCC.

Besides, to confirmed that the β‐catenin was the most regulator of FGF9 mediated ECM production, we used XVA939 to block the β‐catenin signaling pathway. The XAV939 treatment exhibited significant inhibition of FGF9‐induced tumorigenesis both in vitro and in vivo studies. However, XAV‐939 was noted to significantly suppress the tumor progression when mono‐treatment in Xenograft model study. We speculate that it was due to the high expression level of β‐catenin in tumor cells,^[^ ^46^, ^47^, ^48^ ^]^ and that XAV‐939 treatment disrupted the background expression of β‐catenin. This suggestion was proved in immunofluorescence results, showing decreased β‐catenin level after XAV‐939 treatment. Moreover, except for the effect on degradation of β‐catenin, other signaling pathways might be implied in the inhibited effect of XAV‐939 on FGF9‐mediated tumorigenesis. Therefore, direct inhibition of β‐catenin by using AAV‐mediated knockdown or hepatic knockout mice might be more explicit to demonstrate whether FGF9 functions in a β‐catenin dependent manner. We will further explore this issue in future study.

Interestingly, difference to our conclusion herein that FGF9 aggravated the β‐catenin‐mediated accumulation of ECM, it has been previously reported that activation of β‐catenin results from accumulation of ECM and activation of ECM‐related signaling in several types of tumors.^[^ ^11^, ^49^, ^50^ ^]^ Thus, we suggested that a positive feedback loop linking β‐catenin activation and ECM accumulation exists, which could constitute a potential carcinogenic mechanism during the development of NASH‐driven HCC. Our results partially support this hypothesis, with high expression of FGF9 and β‐catenin strongly associated with severe fibrosis in NASH‐driven HCC models with ECM accumulation. Thus, further study is needed to address the regulatory network between FGF9 and β‐catenin, which could be important for the carcinogenic signaling involved in NASH‐driven HCC initiation and progression.

FGF9 has already been reported to enhance the proliferation, aggressiveness and migration of HCC cells in vitro.^[^ ^30^, ^32^ ^]^ Similarly, our study also confirmed that FGF9 regulates tumorigenesis in vitro, as shown by the increased mitotic progression, aggressiveness and migration of liver cancer cells. However, increased FGF9 expression in liver alone did not induce tumor formation and hardly mimic the tumor formation in the HFHC diet and CCl_4_ treatment model in our study. Although FGF9 process the activity of promoting proliferation, aggressiveness and migration of HCC cells in vitro, it hardly caused the HCC alone. Like the findings in CCl_4_‐treated mice, FGF9 TG mice feeding HFHC diet also exhibited a dramatic increase in fibrosis and HCC progression. Our findings indicate that FGF9 promotes hepatic ECM accumulation and that increased expression of FGF9 worsens the pathophysiology of NASH and promotes the occurrence of liver cancer. Because FGF9‐induced development of HCC requires a NASH diet, we speculated that additional stimulation induced by the NASH diet is required for the conditions needed for malignant transformation in the liver, such as elevated inflammation, an increased cholesterol level, steatosis and insulin intolerance. Therefore, our study suggests that FGF9 promotes the accumulation of ECM and that liver fibrosis, rather than the proliferation ability, is the key reason for the occurrence of NASH‐driven HCC.

Another issue that needs further research is the role of FGF9 in inflammation during the development of NASH‐driven HCC. Our KEGG enrichment analysis results implied that another possible function of FGF9 in liver cancer is the activation of inflammation, a feature also associated with NASH.^[^ ^51^ ^]^ This possibility is further supported by the expression of inflammatory factors, such as TNF‐α, IL‐6 and IL‐1β, in the livers of FGF9 TG mice. Collagen release from dying hepatocytes appears to stimulate a certain degree of inflammation/fibroblast activation, which is believed to be supplemented by signals from other sources, such as FGF9‐activated cells.

In conclusion, FGF9 contributes to NASH‐mediated induction of hepatocyte ECM signaling to promote HCC. Potential acceleration of the hepatic fibrosis‐promoting effect of FGF9 could occur through positive regulation of hepatocyte β‐catenin signaling to increase ECM production in the liver. The resulting increase in hepatic fibrosis may be a carcinogenic mechanism by which FGF9 promotes NASH‐driven HCC. Our findings highlighted the potential pharmacological targeting of FGF9, particularly for its fibrosis‐promoting effect, as a promising clinical strategy to treat NASH at an early stage and thereby prevent NASH‐driven HCC formation.

## 4 Experimental Section

### Clinical Specimens

Human liver samples of HCC and benign adjacent tissues were obtained from the First Affiliated Hospital of Anhui Medical University (Hefei, China) and the Affiliated Chuzhou Hospital of Anhui Medical University (Chuzhou, China). None of the patients received chemotherapy prior to hepatectomy. The use of clinical HCC specimens was in accordance with the Declaration of Helsinki. This study was approved by the research ethics committee of Anhui Medical University, and written informed consent was obtained from each participant enrolled in this study.

### Animal

C57BL/6J (male; 8 weeks old) and BALB/c nude mice (male) were purchased from GemPharmatech Co., Ltd, China. All mice were maintained on a 12 h dark/light photoperiod with access to a regular unrestricted diet.

FGF9 Rosa26 knock‐in mice were purchased from Beijing Biocytogen Co., Ltd, China. Briefly, to generate FGF9 Rosa26 knock‐in mice, FGF9 cDNA was inserted downstream of the Rosa26 promoter and a loxP‐stop‐loxP cassette. Subsequently, FGF9 Rosa26 knock‐in mice were crossed with Alb‐Cre mice to generate liver‐specific FGF9 transgenic (FGF9^Alb^) mice. FGF9 Rosa26 knock‐in mice were used as controls.

During the knockdown of FGF9, re‐administration of AAV to enable sustained knockdown of FGF9 in the liver was adopted. The AAV‐shFGF9 (2 × 10^11^vp) diluted in sterile saline were injected into the tail vein (300 µL) to specifical knockdown of FGF9 in the liver. The control groups were injected with same dose of AAV‐shCtrl. At the same time of the AAV injection, rapamycin (1.5 mg kg day^−1^; for 5 days) was intraperitoneal injection to modulate AAV immunogenicity and enable effective AAV re‐administration.^[^ ^52^ ^]^ 3 months after first AAV injection, it was re‐administration of AAV to enable sustained knockdown of FGF9 in the liver. All mice were maintained in a specific pathogen‐free environment on a 12 h dark/light photoperiod with access to a regular unrestricted diet. All animal experiments were approved by the Animal Center of Anhui Medical University. The experimental protocols used for animal studies were approved by the Animal Experimental Committee of Anhui Medical University.

### NASH‐Driven HCC Development and Treatment

To establish the NASH‐driven HCC mouse model, 8‐week‐old mice were fed a normal SD or a high‐fat and high‐cholesterol (HFHC) diet containing 60% calories from fat and 0.5% added cholesterol. CCl_4_ (Sigma–Aldrich, 289116‐100ML) at a dose of 0.2 µL g^−1^ body weight, which was much lower than the dose usually given for fibrosis induction with CCl_4_ alone,^[^ ^25^ ^]^ or the corresponding vehicle control, corn oil, was injected intraperitoneally into mice fed above mentioned diets once per week, starting simultaneously with diet administration. Mice were euthanized 6 months after treatment.

For the induction of NASH‐driven HCC in FGF9^Alb^ mice, FGF9^Alb^ mice or FGF9^Rose^ mice were fed a HFHC diet for 8 months. To determine the effect of XAV‐939 treatment on HCC, five months after HFHC feeding, the mice were administered of XAV‐939 (0.4 mM, MCE) or saline (3 times/week) by intraperitoneal injection. Liver from vehicle and XAV‐939 administered mice were collected at 3 months after injection.

At the time of sacrifice, animals were anaesthetized and exsanguinated; in addition, macroscopic tumors were counted. Liver and serum were harvested and processed for subsequent analysis.

### Macroscopic Tumor Quantification

Whole freshly harvested liver was placed on the paper with 5‐milimeter lines, and photographed. Pictures were used to digitally quantify the area of macroscopic tumors in the liver from the pictures from both sides using Image J (NIH). The images were first calibrated using the 5‐mm lines and afterward the positive tumor areas on the liver surface were manually selected, counted and quantified under microscope.

### Xenograft Model

To induce the formation of heterotopic tumors by Huh7 cells, 8‐week‐old athymic nude (immunodeficient) mice (Charles River) were subcutaneously injected with 1 ×10^6^ cells (in 200 µL PBS) stably overexpressing either FGF9 or green fluorescent protein (GFP) in the left or right front lower limb, respectively. Tumors were allowed to grow for 4 weeks.

To examine the effect of XAV‐939 on the FGF9‐induced tumors, the mice were randomly divided into four groups. Eight days after injection of the Huh7 cells mentioned above, mice were injected peritumorally with XAV‐939 (0.2 mM, MCE) or vehicle (corn oil) every other day for 4 weeks.

The subcutaneous tumor volumes and weights were regularly recorded. Tumor volume was calculated as V(mm^3^) = (length) × (width) × (height) × 0.5. After resection, all tissues were preserved by paraffin embedding. Subsequently, these tumor tissues were subjected to pathological staining.

### RNA‐Sequence

Huh7 cells with stable expression of FGF9 were used for RNA‐seq, and the normal cells were used as control. Total RNA was extracted using Trizol reagent (Life) following the manufacturer's protocol. High‐quality RNA samples with RIN number > 7.0 were used to construct sequencing library. RNA‐seq was performed by Illumina NovaseqTM6000 sequence platform (SEQHEALTH, Wuhan, Hubei, China). Genes differential expression analysis was performed by DESeq2 software between two different groups. The genes with the parameter of false discovery rate (FDR) below 0.05 and absolute fold change ≥2 were considered differentially expressed genes (DEGs). These DEGs were then subjected to enrichment analysis of KEGG pathway, GO functions and GEAS analysis. The RNA‐sequencing data had been deposited in NCBI under SRA accession numbers (SRA: PRJNA986957).

### Statistical Analyses

Quantitative data were presented as the means ± SEM. In most in vivo experiments on mice, *n* = 6 was the minimum number of animals used. For cell studies, the data were representative of at least three independent experiments. All statistical analyses were performed using GraphPad Prism (v.6.0c). Statistical significance was defined as *p* < 0.05 and determined by two‐tailed, unpaired Student's *t* test (for comparison between two experimental conditions) or ANOVA (for comparison among three or more experimental conditions), as indicated in the figure legends and otherwise.

## Conflict of Interest

The authors declare no conflict of interest.

## Author Contributions

L.Z., Q.Z., D.T., M.G. contributed equally to this work. H.B.Z., Y.S.C., W.C., Y.G. and L.Z. conceptualized the project. L.Z., Q.Z., D.T., M.Y.G., K.C.T., X.M.Z., D.K.H. and C.P.R. performed the laboratory experiments. D.T., Z.L.W. and D.K.H. were responsible for obtaining the HCC patient samples. L.Z., H.B.Z., Y.G., X.W., X.Y.W., Q.S.Y. and W.J.Z. did the analysis. L.Z. and H.B.Z. provided funding. L.Z., Q.Z., Y.S.C. and H.B.Z. wrote the original draft. All authors read and commented on the final manuscript. H.B.Z. acts as guarantor for the study.

## Supporting information

Supporting Information

## Data Availability

The data that support the findings of this study are available on request from the corresponding author. The data are not publicly available due to privacy or ethical restrictions.
