# Supplementary material for: FGF9 Recruits β‐Catenin to Increase Hepatic ECM Synthesis and Promote NASH‐Driven HCC
Source: Adv Sci (Weinh). 2023 Aug 11;10(28):2301166. doi: 10.1002/advs.202301166 (PMC10558677; doi:10.1002/advs.202301166)
Supplement: Supplementary file 1 — Supporting Information [file ADVS-10-2301166-s001.pdf]

ADVANCED SCIENCE
Open Access

Supporting Information

for *Adv. Sci.*, DOI 10.1002/advs.202301166

FGF9 Recruits β-Catenin to Increase Hepatic ECM Synthesis and Promote NASH-Driven HCC

Lei Zhang, Qing Zhang, Da Teng, Manyu Guo, Kechao Tang, Zhenglin Wang, Xiang Wei, Li Lin, Xiaomin Zhang, Xiuyun Wang, Dake Huang, Cuiping Ren, Qingsong Yang, Wenjun Zhang, Yong Gao, Wei Chen*, Yongsheng Chang* and Huabing Zhang**

SUPPLEMENTARY FIGURES

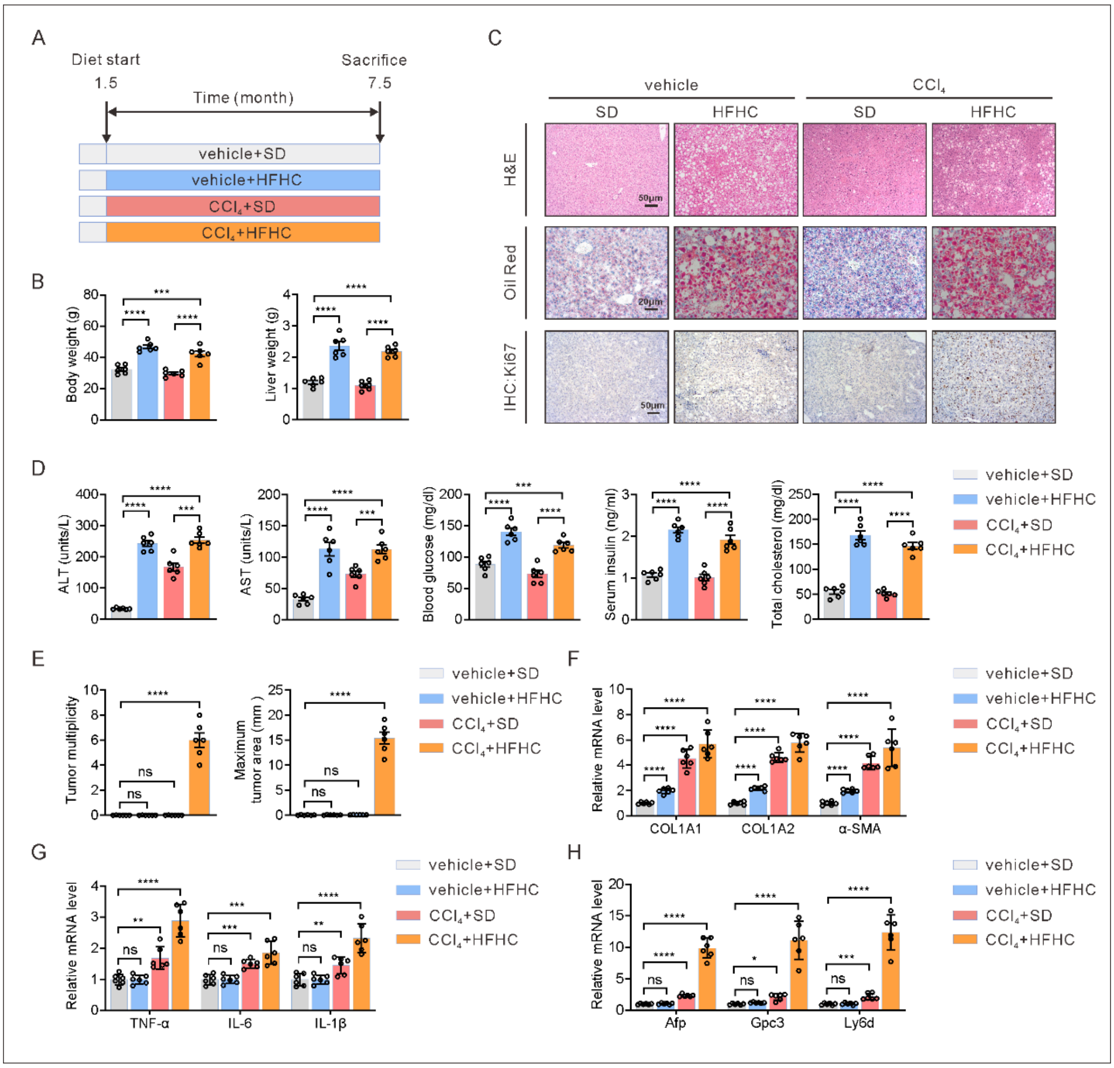

Supplement Figure 1. CCl_4 treatment promotes NASH-driven HCC development in HFHC feeding wild-type mice.

(A) Schematic illustration of the experimental design, with induction of tumourigenesis in liver of mice with treatment with CCl_4 or vehicle once a week at 1.5 month, feeding with SD or HFHC diet for 6 months.

(B) Representative H&E, Oil Red and IHC of Ki67 staining in liver sections from mice fed a SD or HFHC diet and treated with CCl_4 or vehicle.

(C) Body weight and liver weight were measured at 24 weeks.

(D) Serum ALT, AST, blood glucose, plasma insulin, and total cholesterol were measured at 24 weeks.

(E) Quantification of the tumour burden and maximal area in mice fed a SD or HFHC diet and treated with CCl_4 or vehicle.

(F-G) RT-qPCR analysis of the mRNA expression of ECM related genes (F), tumour markers (G) and inflammation-related genes (H) in whole liver tissue.

The data are shown as the means ± SEM. *P < 0.05, **P < 0.01, ***P < 0.005, ****P < 0.001; 2-way ANOVA (C, D, E, F, G, H).

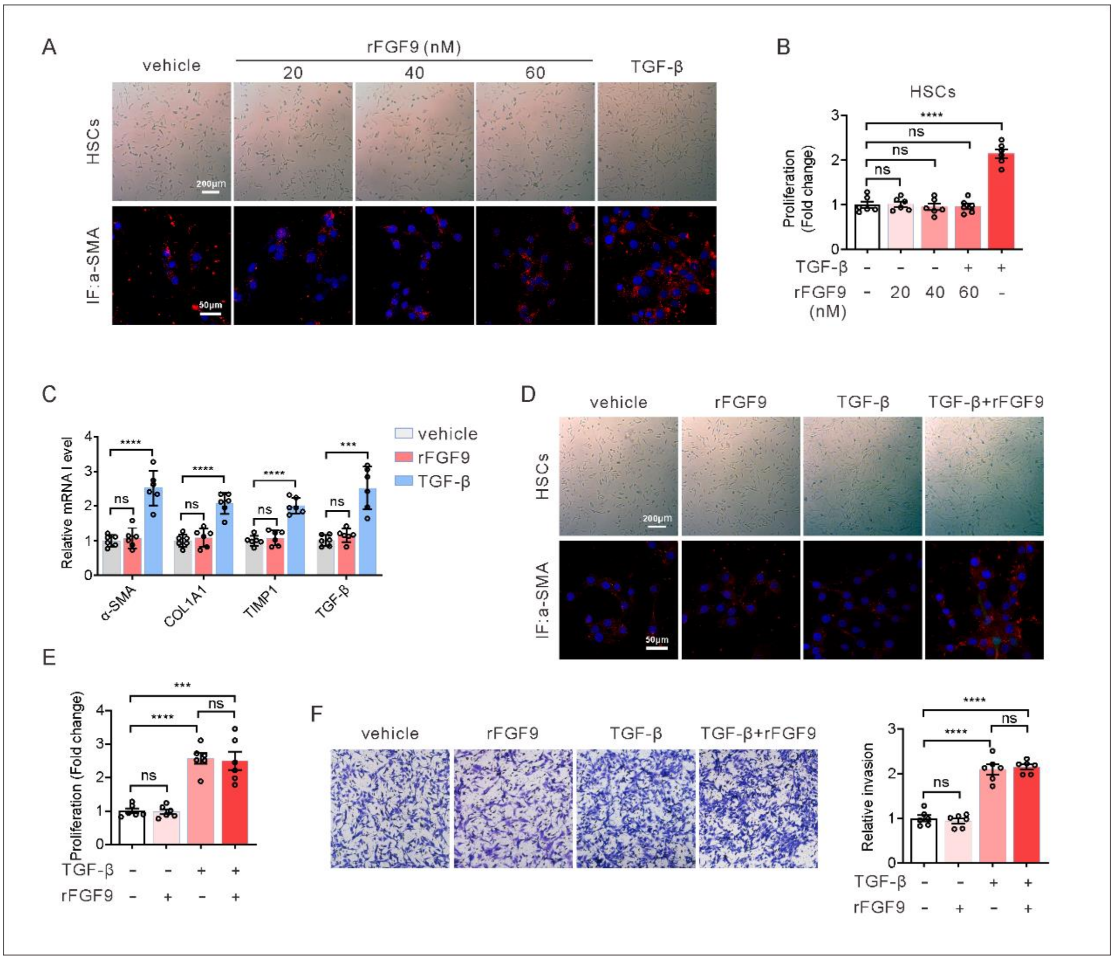

Supplement Figure 2. rFGF9 treatment do not influence the HSCs activation.

(A) Macroscopic images and confocal microscopy analysis of α-SMA of HSCs with the serial doses of rFGF9 treatment.

(B) Cell proliferation of HSCs with the serial doses of rFGF9 treatment.

(C) The mRNA level of α-SMA, COL1A1, TIMP1 and TGF-β of HSCs with the 60 nM rFGF9 treatment.

(D) Macroscopic images and IF analysis of α-SMA of HSCs with 60 nM rFGF9 and TGF-β treatment.

(E) Cell proliferation of HSCs with rFGF9 and TGF-β treatment.

(F) Cell invasion of HSCs with rFGF9 and TGF-β treatment.

The data are shown as the means ± SEM. ***P < 0.005, ****P < 0.001; 2-way ANOVA (B, C, E, F).

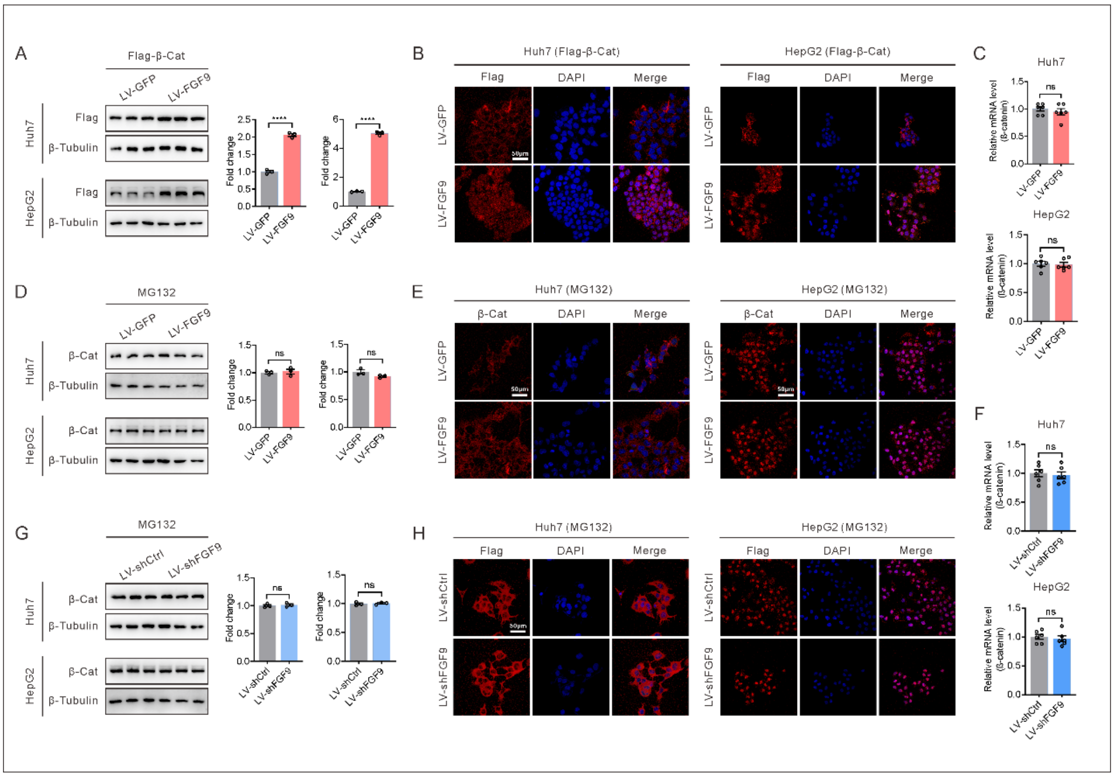

Supplement Figure 3. MG132 blocks the regulation of FGF9 on β-catenin stability.

(A and B) The exogenous β-catenin level in LV-GFP or LV-FGF9 infected Huh7 and HepG2 cells, as examined by Western blot analysis (A) and IF analysis (B).

(C) RT-qPCR analysis of β-catenin in LV-GFP or LV-FGF9 infected Huh7 and HepG2 cells.

(D and E) The effect of MG132 on FGF9 overexpression mediated endogenous β-catenin level in Huh7 and HepG2 cells, as examined by Western blot analysis (D) and IF analysis (E).

(F) RT-qPCR analysis of β-catenin in LV-shCtrl or LV-shFGF9 infected Huh7 and HepG2 cells.

(G and H) The effect of MG132 on FGF9 knockdown mediated endogenous β-catenin level in Huh7 and HepG2 cells, as examined by Western blot analysis (G) and IF analysis (H).

The data are shown as the means ± SEM. ****P < 0.001; 2-tailed Student's t test (A, C, D, F, G).

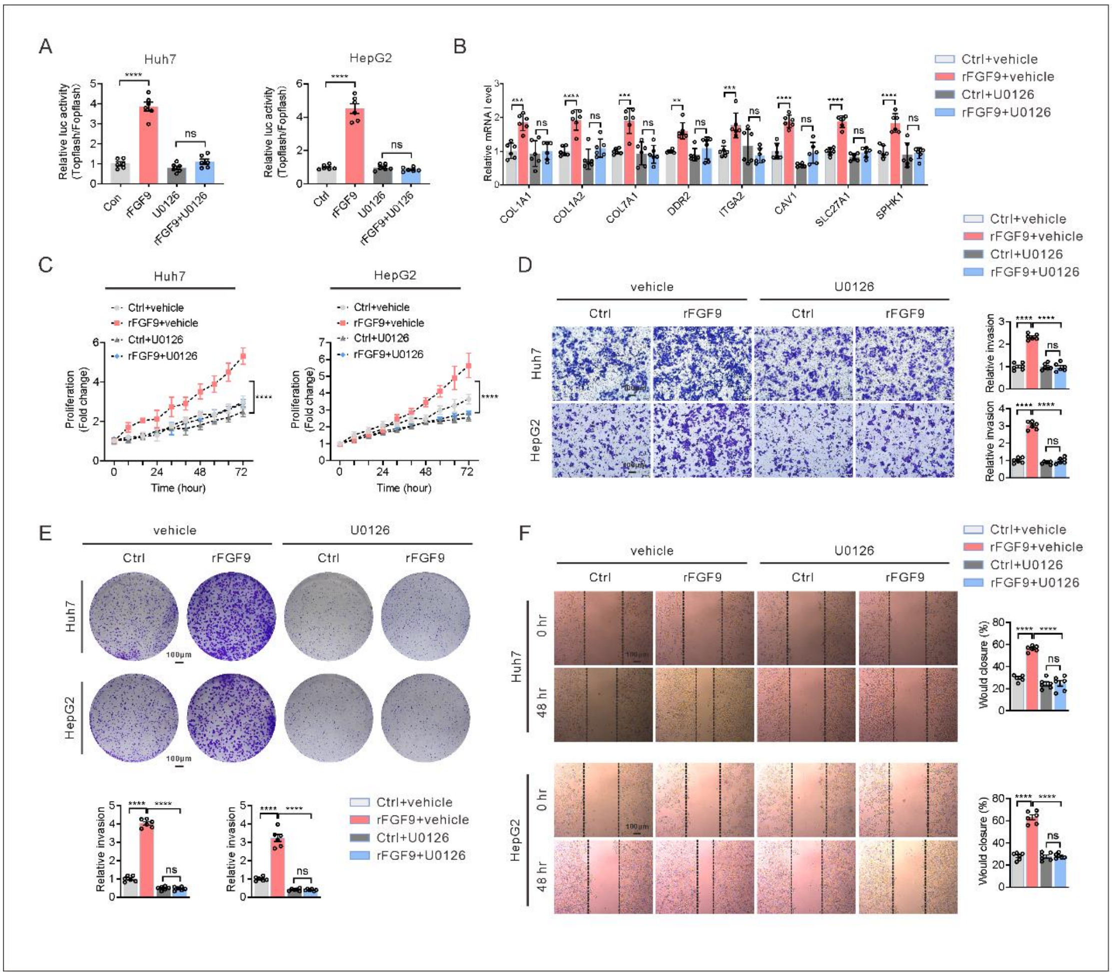

Supplement Figure 4. FGF9 regulates the HCC cells proliferation, migration and invasion via the ERK1/2 pathways in Huh7 and HepG2 cells.

(A) Analysis of changes in TOP-Flash reporter activity induced in Huh7 and HepG2 cells by treatment with rFGF9 and U0126.

(B) RT-qPCR analysis of ECM-related genes in Huh7 cells by treatment with rFGF9 and U0126.

(C-F) Cell proliferation assays (C), colony formation assays (D), invasion assays (E) and migration assays (F) of Huh7 and HepG2 cells treatment with rFGF9 and U0126.

The data are shown as the means ± SEM. ****P < 0.001; 2-way ANOVA (A, B, C, D, E, F).

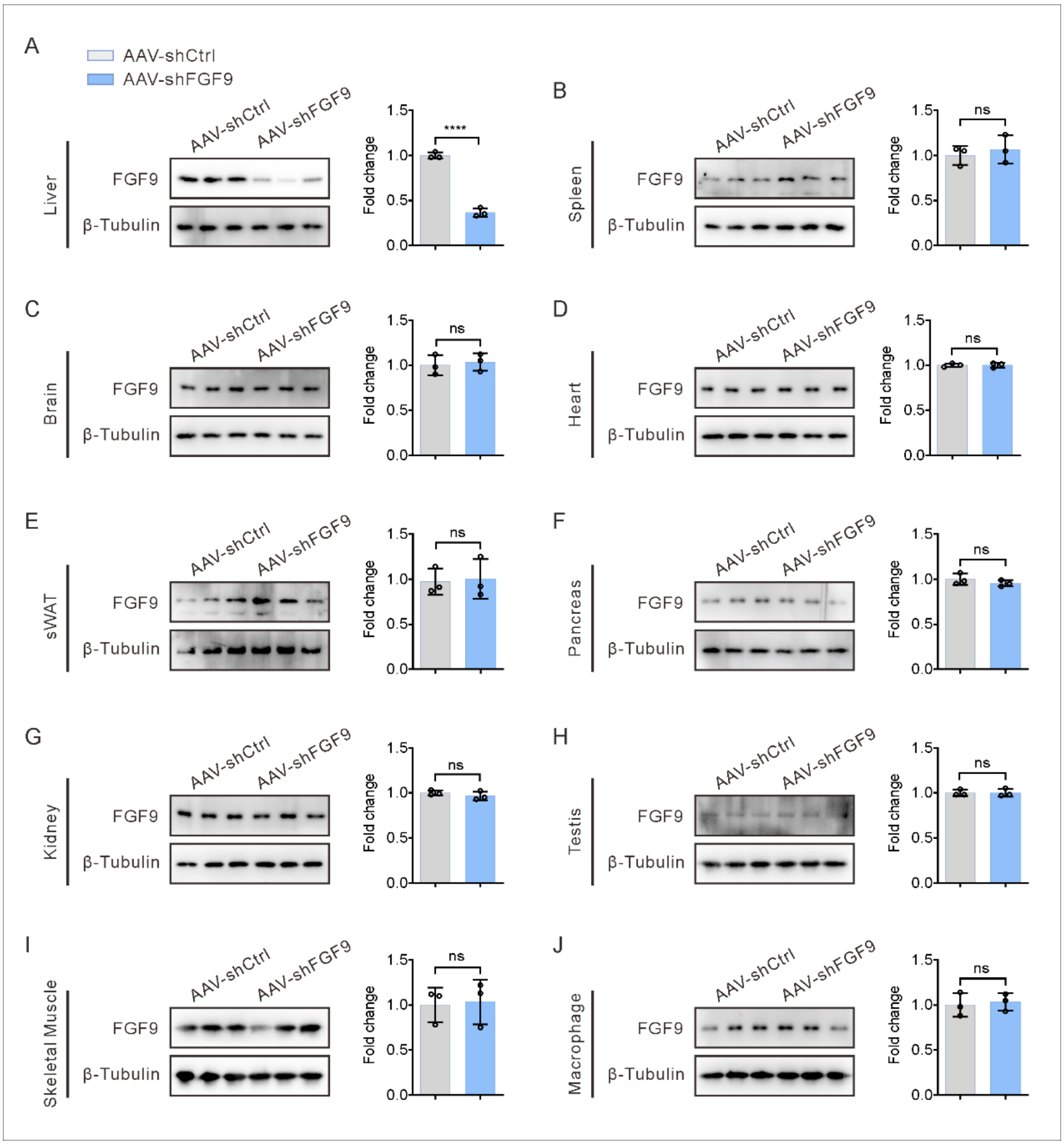

Supplement Figure 5. AAV-shFGF9 decreases FGF9 expression in livers.

(A-J) The protein level of FGF9 in livers and other tissues at 6 months after first tail vein injection of AAV-shCtrl or AAV-shFGF9.

The data are shown as the means ± SEM. ****$P < 0.001$; 2-tailed Student's t test (A, B, C, D, E, F, G, H, I, J).

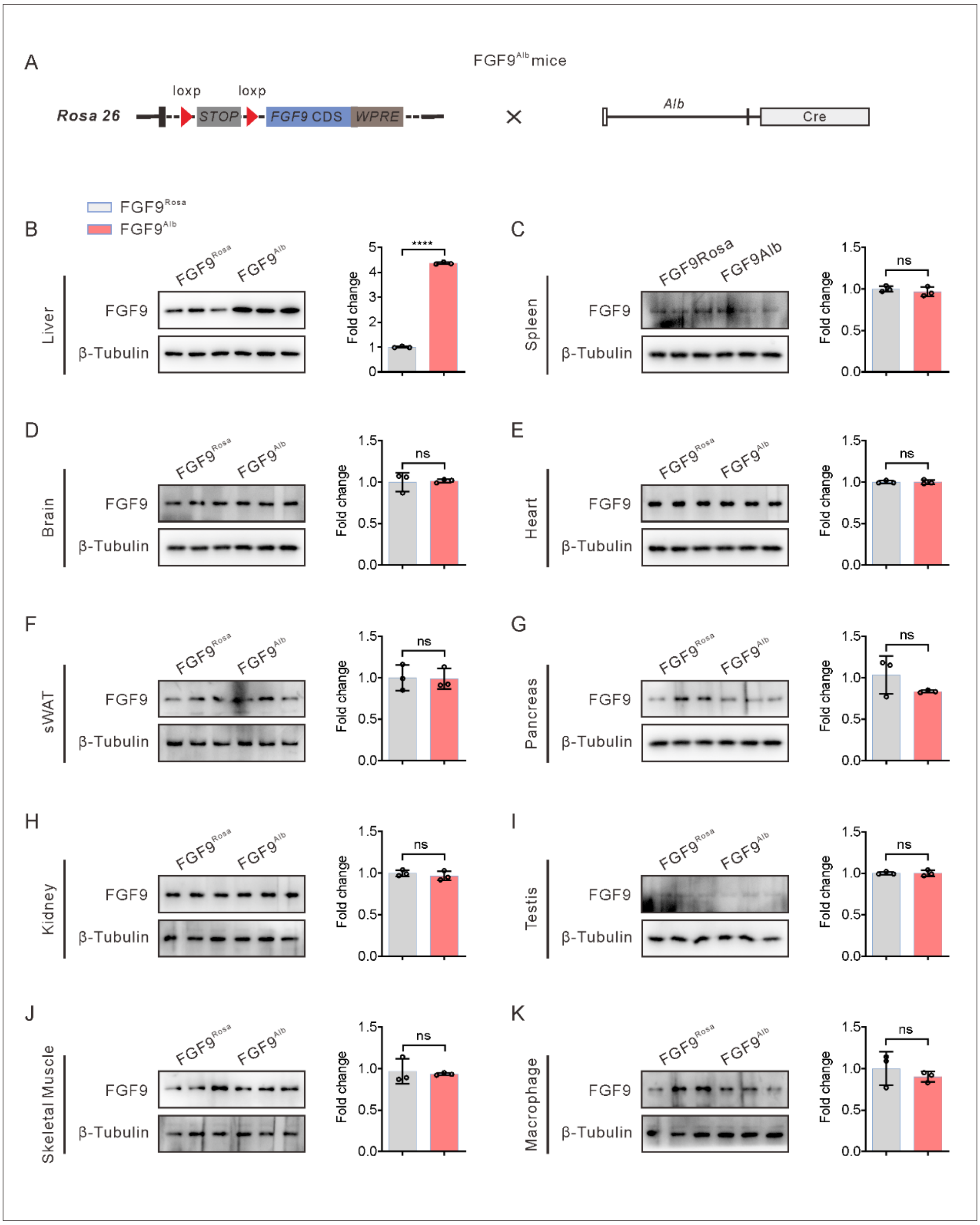

Supplement Figure 6. Generation of hepatic FGF9 transgenic mice.

(A) Schematic illustration of the generation of FGF9 transgenic mice.

(B-K) The protein level of FGF9 livers and other tissues from HFHC diets feeding FGF9^{Rosa} and FGF9^{Alb} mice for 8 months.

The data are shown as the means ± SEM. ****P < 0.001; 2-tailed Student's t test (B, C, D, E, F, G, H, I, J, K).

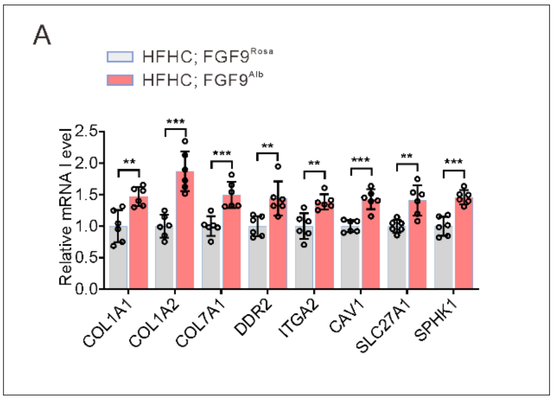

Supplement Figure 7. FGF9 regulates ECM-related genes expression.

(A) The mRNA level of FGF9 in livers 2 weeks from FGF9^{Rosa} and FGF9^{Alb} mice.

The data are shown as the means ± SEM. **P < 0.01, ***P < 0.005; 2-tailed Student's t test (A).

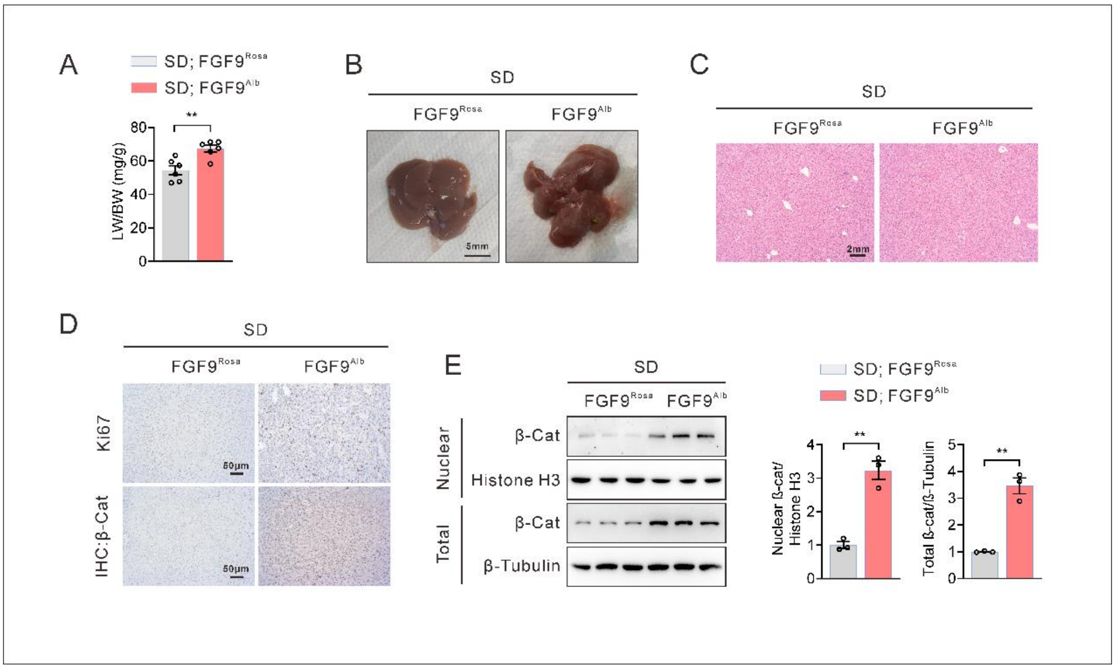

Supplement Figure 8. The phenotypes of FGF9^{Alb} mice fed with SD diet.

(A) Quantitative data for the Liver weight (LW)/Body weight (BW) of FGF9^{Rosa} and FGF9^{Alb} mice fed SD diet for 10 months.

(B and C) Representative macroscopic images (B) and H&E staining (C) in liver sections from FGF9^{Rosa} and FGF9^{Alb} mice fed SD diet for 10 months.

(D) Representative Ki67 and β-catenin staining (IHC) in liver sections from FGF9^{Rosa} and FGF9^{Alb} mice fed SD diet for 10 months.

(E) Nuclear and total β-catenin level, as determined by Western blotting.

The data are shown as the means ± SEM. **P < 0.01; 2-tailed Student's t test (A, E).

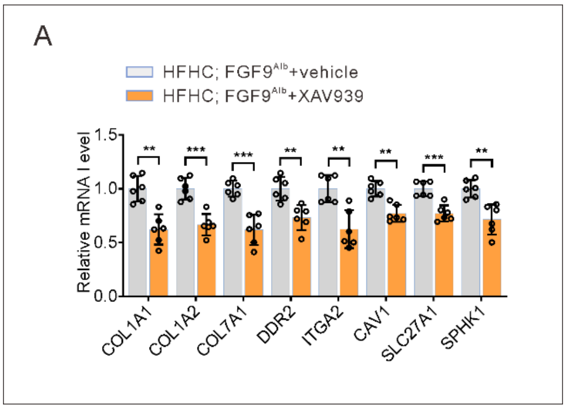

Supplement Figure 9. XAV939 blocks the increased expression of ECM-related genes by FGF9.

(A) RT-qPCR analysis of ECM-related genes in livers from FGF9^{Alb} mice fed HFHC diet and treated with or without XAV939.

The data are shown as the means ± SEM. **P < 0.01, ***P < 0.005; 2-tailed Student's t test (A).

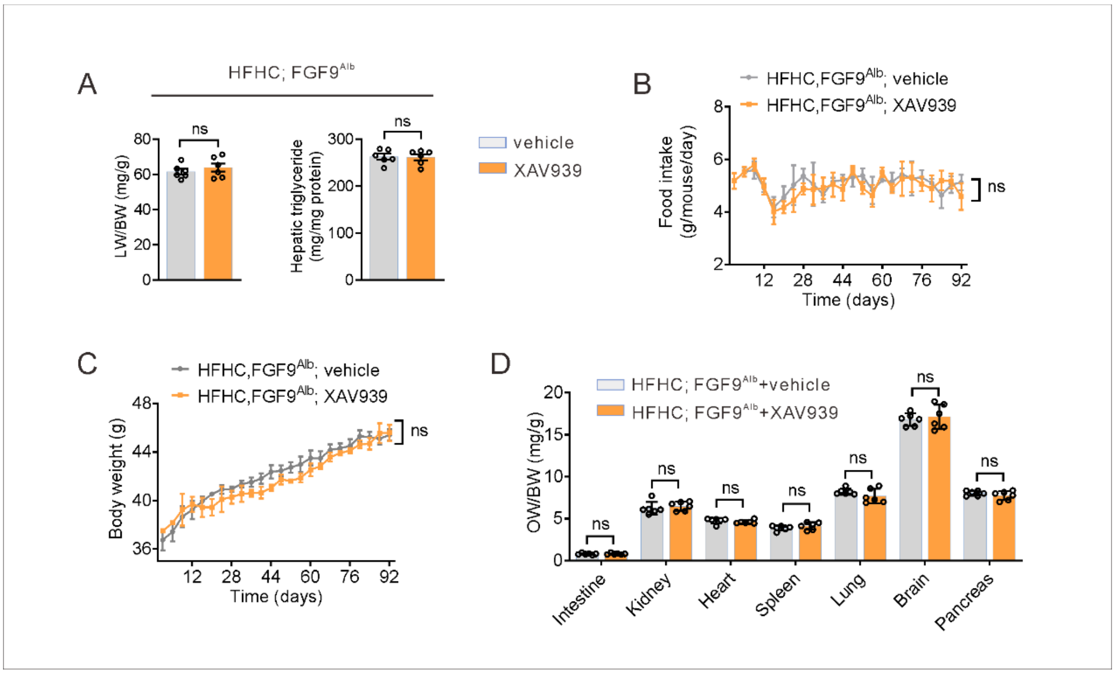

Supplement Figure 10. XAV939 has minimal toxicity for mice.

(A-C) Quantitative data for the LW/BW, liver triglyceride (A), food intake (B) and body weight (C).

(D) Quantitative data for the organ weight (OW)/Body weight (BW) of FGF9^{Alb} mice fed HFHC diet and treated with or without XAV939.

The data are shown as the means ± SEM. 2-tailed Student's t test (A, D), 2-way ANOVA (B, C).

SUPPLEMENTARY METHODS

Immunohistochemistry (IHC) and Immunofluorescence (IF) analysis

Histological studies were performed on mice lives fixed with 4% paraformaldehyde solution in PBS and were subsequently paraffin-embedded. Then, the paraffin lives were sectioned (5 μm) on a microtome, deparaffinized, rehydrated, and pretreated with citrate buffer (10 mmol/L, pH 6.0, Beijing Zhong Shan-Golden Bridge Biological Technology Co. Ltd) for antigen retrieval. Serial liver sections were washed in PBS, and then endogenous peroxidase activity was blocked using an endogenous peroxidase inhibitor (Beijing Zhong Shan-Golden Bridge Biological Technology Co. Ltd.) for 30 min at room temperature. After three washes with PBS, the liver sections were incubated overnight at 4°C with primary antibodies in a humidified chamber. Samples were washed three times and incubated with a secondary antibody (1:100; Solarbio) for one hours at room temperature. Images were acquired with a Leica DM6B microscope.

For tissue immunofluorescence analysis, sections were deparaffinized as described above. After antigen retrieval, the sections were sealed with goat serum (Solarbio) and incubated with primary antibodies followed by FITC-labelled secondary antibodies (1:100; ABclonal). Finally, the sections were stained with DAPI (Solarbio).

For cell immunofluorescence analysis, cells were plated in 24-well plates (2×10^4 cells per well). After incubation for 24-48 h, the cells were fixed on a climbing slide using 4% paraformaldehyde and treated with 0.5% Triton X-100 (Beyotime) in PBS. Then, the cells were incubated with goat serum for blocking. The other steps were similar to those described above for tissue immunofluorescence analysis. Images were acquired with a Leica DM6B microscope.

The primary antibodies used are listed in Table 1.

Supplemental Table 1. Antibodies used in IHC analysis.

FGF9	Abcam; ab115821; 1:100
β-catenin	Abcam; ab32572; 1:100
COL1A1	Abcam; ab270993; 1:100
HRP-conjugated Goat Anti-Rabbit IgG	Abclonal; AS014; 1:500
FITC-conjugated Goat Anti-Rabbit IgG	Abcam; ab6717; 1:500
Alexa-conjugated Goat Anti-Rabbit IgG	Abcam; ab150078; 1:500

H&E staining

Histological studies were performed on mice hearts fixed with 4% paraformaldehyde solution in PBS. Tissues were paraffin-embedded and sectioned into of 5-μm sections. Serial liver sections were stained with hematoxylin and eosin (H&E).

Sirius Red Staining

For tissue Sirius red staining, sections were deparaffinized as described above. The sections were incubated with iron haematoxylin and treated with Sirius red stain. Images were acquired with a Leica DM6B microscope.

Lentiviral packaging and stable cell line construction

The Huh7 and HepG2 cell lines was used to construct stably expressing FGF9-overexpressing and -knockdown cells. For protein overexpression, the PCDH-puro plasmid was cotransfected with psPAX2 and VSV-G at a 4:3:2 mass ratio. Transfection of HEK293T cells was performed using jetPRIME (Polyplus Transfection) according to the manufacturer's protocol. Then, 36 h and 72 h after transfection, virus particles were collected and filtered through a 0.45 μm filter. After using 5× PEG8000 to concentrate the virus, we infected the target cells. Stably transfected cells were obtained after selection in culture medium containing 4 μg/mL puromycin for 2 weeks (Solarbio).

Cell culture and treatment

HEK293T cell line, human HCC cell lines HepG2 (HB-8065) and Huh7 were purchase from the American Type Culture Collection (ATCC). All cells were routinely maintained in DMEM (Gibco) supplemented with 10% foetal bovine serum (Lonsera) 100U/mL and 1% penicillin–streptomycin (Beyotime) and were incubated at 37°C in a humidified atmosphere with 5% CO_2.

Huh7 and HepG2 cells were stimulated with 200 nM rFGF9 (Novoprotein, C198). In the protein degradation assays, protein synthesis was inhibited by the addition of CHX (Biovision) to cells at a final concentration of 50 μg/ml over a time course before harvesting, as indicated in the text. Proteasome activity was inhibited by treating cells with MG132 (Sigma-Aldrich) at a final concentration of 400 μM for 6 hr before cell harvesting. Prior to inhibiting ERK1/2 activity, cells were treated with 10 μM U0126, an ERK1/2 inhibitor (MCE), for 1 hr. To inhibit Wnt/β-catenin pathway, Huh-7 and HepG2 cells were treated with 20 μM XAV939 (MCE).

Cell proliferation and colony formation assays

Cell proliferation was evaluated with MTT (Solarbio) reagent. Huh7 or HepG2 cells were plated in 96-well plates (3×10^3 cells per well), and the absorbance was measured at different time points, as indicated in the text. After indicated operation, 25μL of MTT solution (5 mg/mL) was added to each plate well. After incubation for1 h, 100 μL of DMSO (BioFroxx) was used to dissolve the purple formazan crystals. The absorbance at 490 nm was measured using a microplate reader.

For the colony formation assay, cells were plated in 6-well plates (3×10^3 cells per well) and grown for 10 to 14 days. Then, colonies were washed with PBS, fixed with 4% paraformaldehyde (Sigma) for 20 min, and stained with 0.25% crystal violet (Beyotime) for 30 min at room temperature and photographed under a microscope

Transwell migration assays

Huh7 or HepG2 cells were seeded in 24-well Transwell plates (8.0 µm pore size; Corning) for these assays. For the migration assay, cells were digested and resuspended (5×10^4 cells) in FBS-free DMEM in the upper chamber. Then, 600 µL of culture medium containing 10% FBS was added to the lower chamber. For the invasion assay, we used Transwell membranes precoated with 50 µL of Matrigel (1:6 dilution; BD) and performed the experiment as described above. After 48 h, the cells in the upper chambers were washed with PBS, fixed with 4% paraformaldehyde for 20 min, and stained with 0.25% crystal violet for 30 min at room temperature. Then, the transmigrated cells were photographed using a microscope.

Wound healing assay

Huh7 or HepG2 cells were seeded into 6-well plates at a density of 5×10^5 cells per well. Wound was generated by scratching across the cell monolayer with sterilized pipette tip and the cellular debris was washed away with PBS. Cell migration or protrusion from the wound boarder was measured and photographed under inverted microscope at 0 and 48 h respectively.

RNA extraction and RT-qPCR analysis

After various treatments, total RNA was isolated from cells or tissues using TRIzol reagent (Life). Extracted RNA was reverse transcribed into cDNA with ToloScript RT with DNA Erase-Out (TOLOBIO). The expression levels of mRNA were determined by real-time PCR using SYBR Green (TOLOBIO) according to the manufacturer's instructions. All mRNA expression levels are presented relative to GAPDH mRNA levels. All primer pairs used for qPCR are listed in Table 2.

Supplemental Table 2. Quantitative-PCR primer sequences.

Gene symbol	Primer sequence
Mouse-FGF9	5'- TCCGGTGTTGCTAAGTGACC -3'
	5'- CCGCCTGAGAATCCCCTTTA -3'
Mouse-COL1A1	5'- AGCACGTCTGGTTTGGAGAG -3'
	5'- GACATTAGGCGCAGGAAGGT-3'
Mouse-COL1A2	5'- CCCAGAGTGGAACAGCGATT-3'
	5'- ATGAGTTCTTCGCTGGGGTG -3'
Mouse-COL6A2	5'- ATCCCCCTGATCCAGCAGTT -3'
	5'- TCGACCCCTGTGGAGAACTT -3'
Mouse-COL7A1	5'- ACTGTGGTTGCCCTCTATGC -3'
	5'- TAGCACCTGGTACCCTCCTC -3'
Mouse-COL11A2	5'- CGCCTCCTTCTGTTCCTACC -3'
	5'- GAACACCATCGGGAAGGGAG -3'

Mouse-COL22A1	5’- TGGGGAACCTGGATACGCTA -3’
	5’- AAAGTACGCACACTGGGAGG -3’
Mouse-COLQ	5’- CCATCGTGTCTCAGCCAACT -3’
	5’- GTAGGCAGCATGCTTTGTGG -3’
Mouse-LAMC2	5’- GCACCTGCAAAACTCCACTG -3’
	5’- CCCGTAACTCACTTGCTGGT -3’
Mouse-LAMB3	5’- GCCAGTGTGTGTGCAAAGAG -3’
	5’- TACTGCAGTCACAGGCATGG -3’
Mouse-ITGA2	5’- AGGGTACCATTCGCACCAAG -3’
	5’- CACCATAGCCATCCAGGGAC -3’
Mouse-ITGA3	5’- TGGAGCCCATGTTCAGAGAG -3’
	5’- ATAGTCATCACCCACAGCGAG -3’
Mouse-DDR1	5’- TGGTGGGCCTGGATGATTTC -3’
	5’- GTCTGGAAGGTCCTCAACCG -3’
Mouse-DDR2	5’- GGAGCTGTTGGGTACAGCAT -3’
	5’- TAGCACTTTCGTTCCGCCAT 3’
Mouse-TNC	5’- AAACCATCAGTACCACGGCT -3’
	5’- CATTCTCCGATGCCGTCCAG -3’
Mouse-COMP	5'- AGTTGGCTACATCAGGGTGC -3’
	5'- CGCAGGTTAGCCCAGATGAT -3’
Mouse-CAV1	5’- GACCCCAAGCATCTCAACGA -3’
	5’- TGAAGCTGGCCTTCCAGATG -3’
Mouse-SLC27A1	5’- CGCCGATGTGCTCTATGACT -3’
	5’- ACACAGTCATCCCAGAAGCG -3’
Mouse-SPHK1	5’- CATGAGGTGGTGAATGGGCT -3’
	5’- CCTGCTCGTACCCAGCATAG -3’
Mouse-LRG1	5’- ATGCCTGATACTGCAGTCGG -3’
	5’- GCTGCGTCAGGTTGGAGAAT -3’
Mouse-LIF	5’- GGTGGAGCTGTATCGGATGG -3’

	5’- ATTGAGCTTGACCTGGAGGC -3’
Mouse-36B4	5’- GAGGAATCAGATGAGGATATGGGA -3’<br>5’- AAGCAGGCTGACTTGGTTGC -3’
Human-FGF9	5’- ATGCGGTACCGTTTGGGAAT -3’<br>5’- TGATCCAAGTCCGTGACTGC -3’
Human-COL1A1	5’- GCTCGTGGAAATGATGGTGC -3’<br>5’- ACCCTGGGGACCTTCAGAG -3’
Human-COL1A2	5’- ACAAGGCATTCGTGGCGATA -3’<br>5’- ACCATGGTGACCAGCGATAC -3’
Human-COL6A2	5’- CCAGACCTTCCCTGCCAAAC -3’<br>5’- GCCTTGTGGAAGTTCTGCTC -3’
Human-COL7A1	5’- TGCCTGGTATCCGAGGAGAA -3’<br>5’- TCTCTCCATCAAGGCCACAG -3’
Human-COL11A2	5’- CTCCACAGCAGCAACCATCAA -3’<br>5’- TTCCCTCTCTGGGGTGTGCT -3’
Human-COL22A1	5’- AGGGCAATGTGTTAAGGGGG -3’<br>5’- CTTTGTCTCCTGGTGGTCCG -3’
Human-COLQ	5’- GCGTTCTTCCAATCTCAGCAG -3’<br>5’- CTGGGGAGAGAAGCGGACTT -3’
Human- LAMC2	5’- CATTAGACGGCCTCCTGCAT -3’<br>5’- CGCAGTTGGCTGTTGATCTG -3’
Human-LAMB3	5’- CTTTGACCCCGCTGTGTTTG -3’<br>5’- CTGACACCGCTCACAGTTCT -3’
Human-ITGA2	5’- GTGGCTTTCCTGAGAACCGA -3’<br>5’- GATCAAGCCGAGGCTCATGT -3’
Human-ITGA3	5’- TGTAGGAAGCCCCCTCAAGT -3’<br>5’- ATACAGCAGCCACTTGCCAT -3’
Human-DDR1	5’- TATCCTCATCAACAACCGCCC -3’<br>5’- CAGGTGCACCAGAGCCATT -3’

Human-DDR2	5’- TTGACCGATGGTGTGTCTGG -3’<br>5’- GTAGCCATTGGTGGCACTCT -3’
Human-TNC	5’- TCTGATGGGGGTGGATGGAT -3’<br>5’- CTTCTCTGCGGTCCCCAAAT -3’
Human-COMP	5’- CAGAAGAGCAACCCGGATCA -3’<br>5’- TGTCCCGAGAGTCCTGATGT -3’
Human-CAV1	5’- TGGGGCATTTACTTCGCCAT -3’<br>5’- TAGACACGGCTGATGCACTG -3’
Human- SDC1	5’- CCCTCCACTTGGAAGGACAC -3’<br>5’- CAGGACTACAGCCTCTCCCT -3’
Human-SLC27A1	5’- GTACCACTCGGCAGGAAACA -3’<br>5’- ACTTGATGCAGTCGTCCCAG -3’
Human-SPHK1	5’- GAGAAGTATCGGCGTCTGGG -3’<br>5’- CTACAGGGAGGTAGGCCAGT -3’
Human-LRG1	5’- TGGATCTAACCCGAAACGCC -3’<br>5’- CAGATGCCCCAGAGCTTTCA -3’
Human-LIF	5’- GTCTTGGCGGCAGTACACAG -3’<br>5’- CGACTATGCGGTACAGCTCC-3’
Human-GAPDH	5’- ATTCCATGGCACCGTCAAGG -3’<br>5’- TGGACTCCACGACGTACTCA -3’

Western blot analysis

Total protein was extracted from tissues or cells using RIPA lysis buffer (Beyotime) containing 1% protease inhibitor and 1% phosphatase inhibitor. Briefly, samples were loaded in 10% or 8% SDS–PAGE gels for electrophoresis, and proteins were transferred onto polyvinylidene fluoride (PVDF; Millipore) membranes. Then, the membranes were blocked for 1 h with 5% skimmed milk at room temperature followed by incubation with primary antibodies overnight at 4 °C. The membranes were washed with TBST three times and subsequently incubated with secondary antibodies (goat anti-mouse or goat anti-rabbit) at room temperature for 1 h. Finally, immunoreactions were visualized with chemiluminescence solution. The primary antibodies used are listed in Table 3.

Supplemental Table 3. Antibodies used in Western blot analysis.

FGF9	Abcam; ab115821; (WB)1:500; (IHC)1:100
UB	Abclonal; A3207; 1:500
p-ERK1/2	CST; #9101; 1:500
ERK1/2	CST; #9194; 1:500
p-GSK3β	CST; #9322; 1:500
GSK3β	CST; #12456 1:500
p-β-catenin	CST; #2009 1:500
β-catenin	Abclonal; A11512; 1:500
Flag-Tag	Abclonal; AE005; 1:500
HA-Tag	Abclonal; AE008; 1:100
Axin	Abclonal; A16019; 1:500
Histone H3	Abcam; ab1791; 1:1000
β-Tubulin	Abclonal; AC008; 1:1000
HRP-conjugated Goat Anti-Rabbit IgG	Abclonal; AS014; 1:5000
HRP-conjugated Goat Anti-Mouse IgG	Abclonal; AS003; 1:5000

Immunoprecipitation analysis

Protein complexes were precipitated from whole-cell lysates or different cell fractions with anti-HA or anti-Flag agarose affinity gel (Sigma–Aldrich). The agarose beads were then pelleted by centrifugation and washed three times with lysis buffer. Immunoprecipitates were eluted by boiling the beads in loading buffer. After centrifugation, the supernatants were subjected to SDS–PAGE and Western blotting. Alternatively, immunoprecipitation was performed using an HA Tag or Flag Tag IP/Co-IP Kit (Thermo Scientific) following the instruction manuals. For precipitation of endogenous proteins, specific antibodies were used. Protein complexes were then precipitated with protein G-Sepharose (GE Healthcare), followed by the steps described above.
